# Structural and functional responses of invertebrate communities to climate change and flow regulation in alpine catchments

**DOI:** 10.1111/gcb.14581

**Published:** 2019-03-03

**Authors:** Daniel Bruno, Oscar Belmar, Anthony Maire, Adrien Morel, Bernard Dumont, Thibault Datry

**Affiliations:** ^1^ Instituto Pirenaico de Ecología (IPE‐CSIC) Zaragoza Spain; ^2^ IRSTEA, UR MALY, Centre de Lyon‐Villeurbanne Villeurbanne France; ^3^ Marine and Continental Waters Program IRTA Sant Carles de la Ràpita Spain; ^4^ EDF R&D, Laboratoire National d'Hydraulique et Environnement Chatou France; ^5^ IRSTEA, UR RECOVER, Centre d'Aix‐en‐Provence Aix‐en‐Provence France

**Keywords:** aquatic communities, functional diversity, functional traits, global change, hydrologic alteration, macroinvertebrates, rivers, taxonomic diversity

## Abstract

Understanding and predicting how biological communities respond to climate change is critical for assessing biodiversity vulnerability and guiding conservation efforts. Glacier‐ and snow‐fed rivers are one of the most sensitive ecosystems to climate change, and can provide early warning of wider‐scale changes. These rivers are frequently used for hydropower production but there is minimal understanding of how biological communities are influenced by climate change in a context of flow regulation. This study sheds light on this issue by disentangling structural (water temperature preference, taxonomic composition, alpha, beta and gamma diversities) and functional (functional traits, diversity, richness, evenness, dispersion and redundancy) effects of climate change in interaction with flow regulation in the Alps. For this, we compared environmental and aquatic invertebrate data collected in the 1970s and 2010s in regulated and unregulated alpine catchments. We hypothesized a replacement of cold‐adapted species by warming‐tolerant ones, high temporal and spatial turnover in taxa and trait composition, along with reduced taxonomic and functional diversities in consequence of climate change. We expected communities in regulated rivers to respond more drastically due to additive or synergistic effects between flow regulation and climate change. We found divergent structural but convergent functional responses between free‐flowing and regulated catchments. Although cold‐adapted taxa decreased in both of them, greater colonization and spread of thermophilic species was found in the free‐flowing one, resulting in higher spatial and temporal turnover. Since the 1970s, taxonomic diversity increased in the free flowing but decreased in the regulated catchment due to biotic homogenization. Colonization by taxa with new functional strategies (i.e. multivoltine taxa with small body size, resistance forms, aerial dispersion and reproduction by clutches) increased functional diversity but decreased functional redundancy through time. These functional changes could jeopardize the ability of aquatic communities facing intensification of ongoing climate change or new anthropogenic disturbances.

## Graphical abstract

We explored the structural and functional effects of climate change in interaction with flow regulation by comparing data collected in the 1970s and 2010s in regulated and unregulated alpine catchments of France. Divergent structural but convergent functional responses were found between free‐flowing and regulated catchments. Although cold‐adapted taxa decreased in both catchments, greater colonization of thermophilic species was detected in the free‐flowing one, resulting in higher species turnover, and increased taxonomic diversity. The spread of new functional features to cope with climate change and the presence of tolerant taxa increased functional diversity but decreased functional redundancy in both catchments.

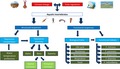

## 1 INTRODUCTION

Understanding and predicting how biological communities respond to ongoing anthropogenic climate change is critical for assessing biodiversity vulnerability and guiding conservation efforts (e.g. Bellard, Bertelsmeier, Leadley, Thuiller, & Courchamp, 2012; Sala et al., 2000). Global circulation models project an increase in air temperatures between 0.3 and 4.8°C before the end of this century depending on regions and climatic scenarios (IPCC, 2013). A stronger increase in air temperature is expected in high‐altitude areas, primarily due to clouds (latent heat release), snow‐albedo and water vapor‐radiative feedbacks and low concentrations of aerosols (Pepin et al., 2015). Furthermore, mountain regions are particularly sensitive to global warming because even a slight increase in air temperature (1–2°C) can cause drastic changes in their biological communities (Cannone, Sgorbati, & Guglielmin, 2007). In this context, glacier‐ and snow‐fed rivers are considered as sentinel systems of climate change and could provide early warning of wider scale changes (Hotaling, Finn, Giersch, Weisrock, & Jacobsen, 2017; Jacobsen, Milner, Brown, & Dangles, 2012; Milner, Brown, & Hannah, 2009; Woodward, Perkins, & Brown, 2010). Alpine catchments are sensitive to the acceleration of glacier retreat and snowpack shrinkage which result in altered meltwater contributions (Brown, Hannah, & Milner, 2007; Milner et al., 2017). Although most significant hydrological changes usually occur in upper areas, seasonal water releases from snow and ice are critical for maintaining water flow, sediment and nutrient transport, as well as ecosystem structure and function, thereby providing vital ecosystem services much farther downstream (Huss et al., 2017). In alpine catchments, the effects of climate change on river communities have been mainly explored at the most upstream reaches, in the vicinity of glaciers and snowpacks (e.g. Brown et al., 2007; Cauvy‐Fraunié et al., 2016; Lencioni, 2018), whereas downstream sub‐alpine river reaches are less frequently considered compared to the upper sections (e.g. Hari, Livingstone, Siber, Burkhardt‐Holm, & Güttinger, 2006; Jasper, Calanca, Gyalistras, & Fuhrer, 2004).

### 1.1 Climate change effects in alpine catchments

Climate change induces modifications in sediment supply and transport as well as in thermal and hydrological regimes, thereby affecting hydrogeomorphological and physico‐chemical conditions downstream (Cauvy‐Fraunié et al., 2013; Hannah et al., 2007; McGregor, Petts, Gurnell, & Milner, 1995). These changes are likely to have cascading effects on the structure and function of aquatic communities (Milner et al., 2017). Reduced sediment loads, warmer water temperature and stabilized channels associated with climate change can lead to significant modifications in species distribution range and contribute to local extinctions (Jacobsen et al., 2014; Milner et al., 2009, 2017; Milner & Petts, 1994). In fact, elevation shifts in species distributions toward higher river reaches, colonization of thermophilic species, increase in the incidence of temperature‐dependent diseases and phenological shifts have been reported for fishes (Hari et al., 2006) and invertebrates (Domisch et al., 2013; Vittoz et al., 2013). Increased temperature and reduced meltwater decreases environmental harshness in upper reaches surrounded by glaciers and snowpacks, which can lead to local taxonomic (alpha diversity) increases (Brown et al., 2007; Milner et al., 2017). Otherwise, these changes can reduce alpha diversity in downstream alpine valleys where glacial and nival influences diminish (Jacobsen et al., 2012). Such changes in alpha diversity can be coupled with decreased between‐site taxonomic differences (beta diversity) due to community homogenization (Cauvy‐Fraunié, Espinosa, Andino, Jacobsen, & Dangles, 2015; Milner et al., 2017), ultimately resulting in a lower regional taxonomic richness (gamma diversity) and loss of endemic species (Brown et al., 2007; Domisch et al., 2013; Jacobsen et al., 2012).

Although functional approaches are still scarce in the context of climate change, changes in taxonomic composition (primarily driven by the different responses of taxa to changes in temperature) could result in shifts in biological traits related to physiology, behavior or dispersal (Bonada, Dolédec, & Statzner, 2007; MacLean & Beissinger, 2017). Climate change (or other anthropogenic disturbances as flow regulation) could alter the organization of functional space through the loss of species with traits poorly adapted to the new environmental conditions and the colonization by better‐adapted species (e.g. more competitive and/or productive species; Mouillot, Graham, Villéger, Mason, & Bellwood, 2013), or by homogenizing the functional structure of aquatic communities (Buisson, Grenouillet, Villéger, Canal, & Laffaille, 2013; Clavel, Julliard, & Devictor, 2011) due to the replacement of specialists by generalist species (Hotaling et al., 2017), which could have deep implications for functional redundancy (FR) and functional diversity (FD) components (functional richness [FRic], functional evenness [FEve] and functional dispersion [FDis]). In addition, there is increasing evidence that dispersal constraints also influence biological responses to climate change. Thus, the species can experience distribution shifts which mainly depend on the capacity to disperse into suitable unoccupied areas. Once individuals of a species disperse, high reproductive potential (i.e. fast life history strategies such as high fecundity, low longevity and small body size) facilitates the establishment of viable populations (Daufresne, Lengfellner, & Sommer, 2009), while the ability to find appropriate food and habitat influences their persistence (MacLean & Beissinger, 2017). Accordingly, generalist species should be more likely to find suitable resources, given their smaller body size, greater diet and habitat breadth (Angert et al., 2011). Such functional homogenization combined with the expected decrease in species richness might involve decreases in FD components and FR, and consequently in ecosystem resilience and resistance (Sonnier, Johnson, Amatangelo, Rogers, & Waller, 2014). In particular, FRic could show a delayed response to climatic change because its decrease requires the loss of species with extreme combinations of traits. By contrast, FDis could diminish as specialist species become locally extinct. Finally, climate change (and other anthropogenic disturbances as flow regulation) is expected to increase the importance of trait filtering (e.g. when original trait modalities are affected in a greater extent than common ones), potentially causing co‐occurring species to become more clustered in functional space, thus decreasing FEve progressively (Mouillot et al., 2013).

### 1.2 Flow regulation effects in alpine catchments

In alpine catchments, harnessing water for the generation of electricity is an exceptionally important factor (Truffer, Markard, Bratrich, & Wehrli, 2001), because water in mountainous regions often constitutes the dominant useful natural resource for electricity production (Wehren, Schädler, & Weingartner, 2010). Thus, many alpine rivers are used for hydropower production, which can cause important hydromorphological alterations such as homogenized habitats, diminished longitudinal connectivity, altered thermal regimes (Caissie, 2006; Dickson, Carrivick, & Brown, 2012; Ward & Stanford, 1983), fine sediment accumulation and streambed clogging (Datry, Lamouroux, Thivin, Descloux, & Baudoin, 2015; Waters, 1995; Wood & Armitage, 1997) as well as altered organic matter and sediment transport (Carlisle, Nelson, & Eng, 2012; Dewson, James, & Death, 2007). Like climate change, these alterations can favor cosmopolitan, non‐indigenous species at the expense of locally adapted native taxa (Poff, Olden, Merritt, & Pepin, 2007). This may reduce taxonomic and functional diversities and compromise the ability of aquatic communities to cope with additional stressors such as climate change (Carlisle et al., 2012; Freedman, Lorson, Taylor, Carline, & Stauffer, 2014; Martínez et al., 2013; Poff & Zimmerman, 2010). Otherwise, particular traits that enable aquatic organisms to deal with hydrological alteration (e.g. multivoltinism, feeding habits; Carlisle et al., 2012) could help them to face warmer temperatures and reduced flow caused by climate change (Statzner & Bêche, 2010). Evidence on the individual effects of climate change and flow regulation on alpine rivers exists; however, there is limited understanding of how dams interact with climate change to alter aquatic communities (Dickson et al., 2012; Palmer et al., 2009; Piggott, Salis, Lear, Townsend, & Matthaei, 2015).

This study addressed the structural and functional effects of climate change and flow regulation on aquatic communities in two alpine catchments in south‐eastern France, one being influenced by dams and intakes of various capacities. Biological responses to climate change and the interaction between both stressors were explored using environmental and biological data collected at 15 sampling sites in the 1970s and compared with those recollected in the 2010s. According to climate change models (IPCC, 2013), both catchments have become drier and warmer in the last few decades. As ecological consequences of these environmental changes between the 1970s and the 2010s, we hypothesized:

1. Decreases in local and regional taxonomic richness (alpha and gamma diversities, respectively), and in the dissimilarity among sites (spatial beta diversity) due to biotic homogenization in both catchments.
2. The replacement of psychrophilic by eurythermic and thermophilic taxa, leading to a high temporal turnover and changes in taxonomic composition patterns.
3. Shifts in functional traits to cope with climate change: reductions of body size and aquatic dispersion, but increase of taxa with resistance forms and several reproductive cycles per year.
4. Declines in community functional redundancy and diversity indices due to shifts in functional traits and biotic homogenization.

We expected such changes to be more pronounced in the regulated catchment than in the free‐flowing one, due to additive or synergistic effects between flow regulation and climate change on aquatic communities.

## 2 MATERIALS AND METHODS

### 2.1 Study area

**Figure 1:**
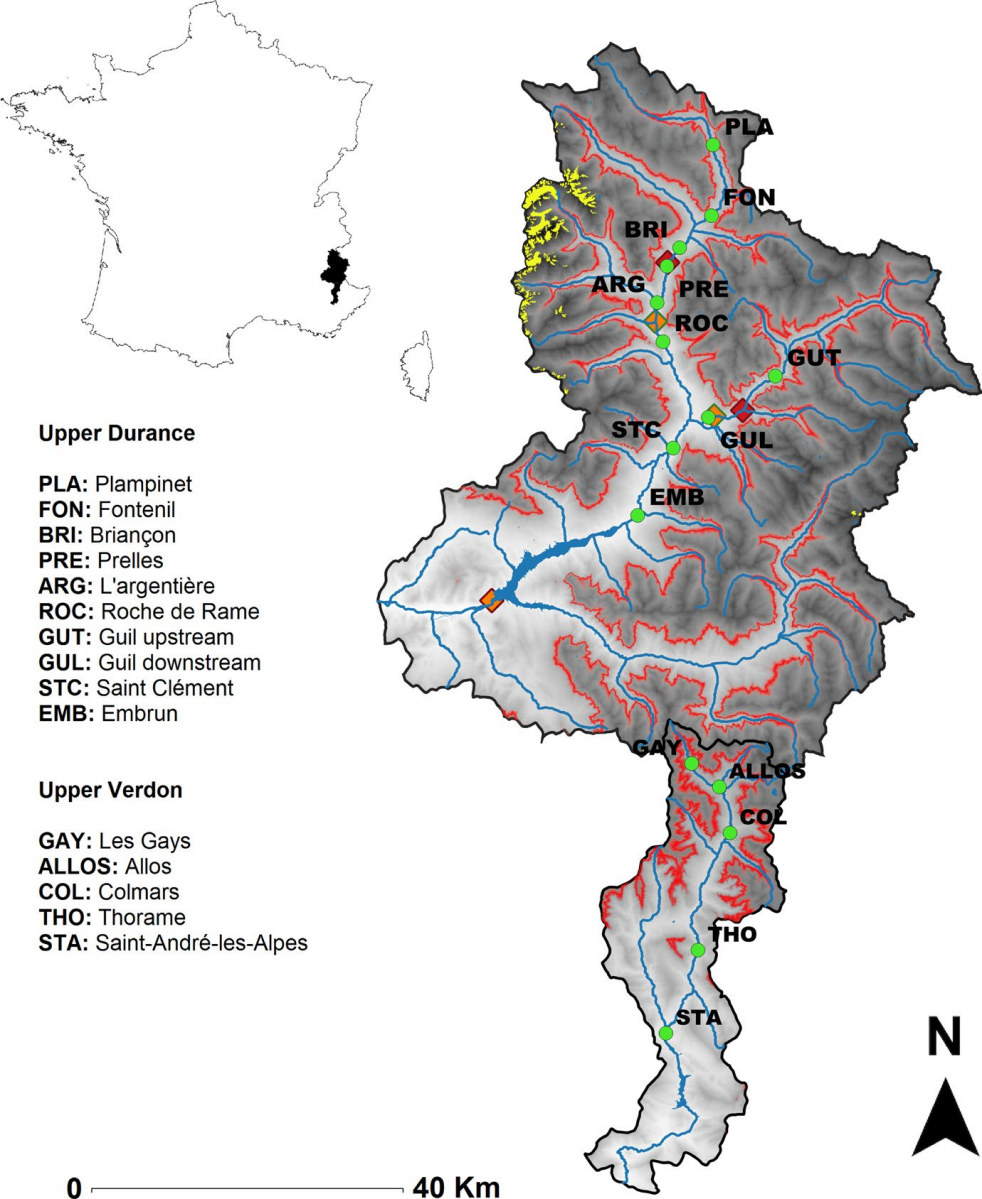
The study area, displaying the location of the catchments (upper Durance—mostly regulated and upper Verdon—free‐flowing), the elevation range (black‐–white gradient from 4,000 to 600 m.a.s.l.), tree‐line (red line at 1,800 m.a.s.l.) and the sampling sites (green dots). Water bodies (rivers, main lakes and artificial reservoirs) and remnant glaciers are shown in blue and yellow color, respectively. Main hydroelectric intakes and outlets are represented by red and orange diamonds, respectively [Colour figure can be viewed at http://www.wileyonlinelibrary.com/]

This study took place in south‐eastern France, in two contiguous alpine catchments (upper Durance and upper Verdon), tributaries of the Rhône River (Figure 1). Both catchments are dominated by sedimentary rocks (limestones, sandstones, dolomites or conglomerates among others) and show a similar range of altitude (from 800 to more than 3000 m.a.s.l.). Snowpack presence is seasonally important in both basins: Durance and Verdon catchments have an average of 170 and 144 days covered by snow (at 1800 m.a.s.l) with a mean depth of 40 and 25 cm, respectively (Durand et al., 2009). In addition, some small (remnant) glaciers (<25 km^2^; Magand, Ducharne, Le Moine, & Gascoin, 2014) are also present in the upper Durance (yellow dots in the left upper section of Figure 1). Climate is similar between both catchments. The upper Durance has a mean annual air temperature of 4.6°C and precipitation of 975 mm, whereas upper Verdon has a mean annual air temperature of 5°C and precipitation of 1125 mm, according to daily time series from 1960 to 2015 from the SAFRAN model (*Système d'Analyse Fournissant des Renseignements Atmosphériques à la Neige* produced by the French Institute of Meteorology; Durand, Giraud, Brun, Merindol, & Martin, 1999). The study area is located in the alpine hydroclimatic region of Southern Alps, which is characterized by glacier, snowmelt and mixed hydrologic regimes (Renard et al., 2008). Natural and seminatural land uses are predominant representing more than 80% of the area for both catchments as estimated from CORINE land‐cover classifications (Coordination of Information on the Environment produced by the European Environment Agency Union; EC, 1993) from 1990 and 2012. Accordingly, although both catchments are scarcely populated, the population of the upper Durance (≈33,000 inhabitants) is notably greater than for the upper Verdon (≈3,000 inhabitants; INSEE, 2014), so more pumping stations, small reservoirs and water diversions are present in upper Durance to meet water demands (mainly due to sky stations, campsites, villages and secondarily agriculture; BD Carthage^®^). There is also a striking difference in terms of the presence of hydropower facilities and weirs between both catchments. Indeed, only the upper Durance is regulated by hydropower installations and diversions, the upstream dams being Argentière (built in 1909), Pont‐Baldy (1963) and Maison du Roi (1979).

### 2.2 Biological data

#### 2.2.1 Invertebrate sampling design

Benthic invertebrates show globally consistent responses to climate change effects (Brown et al., 2018) so they were used as model aquatic communities. Samples were collected in late winter (February) and late summer (August–September) on 15 sites within two catchments in the late 1970s (1977–1979) and recollected in the 2010s (2013–2014) following the same sampling protocol. Sites were distributed longitudinally along both river networks covering an altitudinal range from 800 to 1600 m.a.s.l (sub‐alpine; Figure 1, Table S1.1 in Appendix S1). Five sites were located in the upper Verdon (free‐flowing catchment; hereafter FC) and 10 in the upper Durance (regulated catchment; hereafter RC). In both seasons of the 1970s and the 2010s, two Surber samples (area: 0.1 m^2^; mesh size: 250 µm) were collected from each of the two zones stratified by water velocity: the edge (between 0 and 30 cm/s) and the channel center (>30 cm/s; sampled area per site and season: 0.4 m^2^) in FC. RC sites had a bigger wetted perimeter than FC ones so two extra samples were performed in a third zone to obtain a good spatial representation (two Surbers of 0.1 m^2 ^in three zones: 0–30; 30–80; >80 cm/s; sampled area per site and season: 0.6 m^2^). Invertebrates were preserved in 4% formaldehyde solution and identified in the laboratory. They were sorted, counted and identified to the lowest practical taxonomic level. Most taxa were identified to genus‐ or species‐level except for Diptera, which were identified to family, subfamily or tribe (Chironomidae) and genus when possible. Abundances were standardized by sampling effort to obtain density (calculated as the total number of individuals observed divided by the sampled area) in order to make both catchments comparable. Finally, we averaged seasonal data to obtain annual taxonomic composition and density data for each sampling site and for each time period (data available in Table S1.2 and Appendix S2).

#### 2.2.2 Functional traits

The functional features of invertebrate communities were characterized using 11 biological traits describing life‐history (body size, life‐cycle duration, number of cycles per year and types of aquatic stages), resilience or resistance potentials (dispersal, locomotion and substrate relationship, resistance forms), reproduction, respiration, food preference and feeding habits (Tachet, Richoux, Bournaud, & Usseglio‐Polatera, 2010). These traits are likely responsive to climate change (Bonada et al., 2007). Furthermore, food preference, feeding habits, body size, dispersal and locomotion can be also considered functional effect traits since they represent biological features that directly influence a specific ecosystem function (Hevia et al., 2017). In addition, an ecological trait, called water temperature preference (Tachet et al., 2010), was also analyzed to track potential changes in psychrophilic, eurythermic and thermophilic taxa. Each taxon was coded according to its affinity with each trait category using a fuzzy coding approach (Chevenet, Dolédec, & Chessel, 1994). Fuzzy coding data were then converted to percentages of affinity for each trait. This procedure standardizes the potential differences in the codification scores (i.e. different row sums for each taxon and trait).

### 2.3 Climate and discharge data

For all sampling sites, daily climatic data were obtained for the preceding 20 years including the collection dates, 1960–1979 for the 1970s and 1996–2015 for the 2010s, using the SAFRAN model (Durand et al., 1999; a detailed description of SAFRAN and its applications over France can be found in Quintana‐Seguí et al., 2008). We selected and compared these periods as an acceleration of climate change has been observed globally (NOAA, 2015) and particularly in the Alps from the 1980s (Hari et al., 2006). More specifically, total (mm) and solid (mm) annual precipitation, daily mean (°C), maximum (°C) and minimum (°C) air temperature, annual evapotranspiration (mm), soil humidity (dimensionless index ranging from 0 to 1) and snow water equivalence (mm) were gathered for each sampling site and averaged for each period of 20 years. Similarly, daily and hourly flow data were collected from *Eaufrance* (the French public service on water information; http://www.eaufrance.fr) and *Électricité de France* (EDF) databases. There were only five sampling sites corresponding to gauging stations with enough flow records to hydrologically characterize both time periods (1960–1979 and 1996–2015; Kennard, Mackay, Pusey, Olden, & Marsh, 2010), four located in RC: Fontenil (FON), Briançon (BRI), l'Argentière (ARG) and Embrun (EMB), and one in FC near Saint‐André‐les‐Alpes (STA; Figure 1).

### 2.4 Data analysis

#### 2.4.1 Changes in climatic and flow conditions between the 1970s and 2010s

Changes in each climatic variable between both periods and across catchments were tested using linear mixed‐effect models (LMEs), considering date and catchment (and the interaction between them) as fixed factors and site as random factor (hereafter LME procedure). Goodness of fit was evaluated with Marginal *R* ^2^ associated to fixed effects in LME procedure. Beforehand, Mann–Whitney *U* test and boxplots were performed to verify the temporal representativeness of biological samples for the 1970s (1960–1979) and the 2010s (1996–2015). Thus, we checked whether the total precipitation and mean air temperature of years with biological samples (i.e. 1977–1979 in the 1970s and 2013–2014 in the 2010s) differed from those without biological samples within each 20 year period. Changes in flow regime were assessed by measuring the temporal variations of 45 hydrological indices (Tables S1.3 & S1.4 in Appendix S1) related to flow magnitude and variability selected from Olden and Poff (2003) and computed using the five gauging stations with flow series with a minimum of 15 year records (Kennard et al., 2010) for both periods of time (with the exception of STA in the free‐flowing catchment‐FC, where 12 year records were used for 1970s calculations; Risley, Stonewall, & Haluska, 2008).

#### 2.4.2 Structural changes in invertebrate communities between the 1970s and 2010s

To test spatial and temporal differences in taxonomic diversity (Hypothesis 1), indexes describing the different components of aquatic invertebrate diversity (*α*, *β* and *γ*) were computed. First, *α*‐diversity was calculated as the taxonomic richness of each sampling site. “LME procedure” was performed to check differences in *α*‐diversities between dates (1970s and 2010s) and catchments (RC‐regulated and FC‐free flowing). Second, *β*‐diversity (*β*SOR, Sørensen's dissimilarity) and spatial turnover in taxonomic composition (*β*SIM, Simpson's dissimilarity) were calculated using occurrence data (Baselga & Orme, 2012). Then, whether sampling sites had higher *β*SOR and *β*SIM in FC than those located in RC were explored through null models for each period, which accounted for the different number of sites between catchments (Zamora‐Marín, Gutiérrez‐Cánovas, Abellán, & Millán, 2016). For this purpose, we estimated the spatial *β*SOR and *β*SIM among sites located in FC and compared them to expected values from 999 random draws of an equal number of sites, taken from the pool of those located in RC. The proportion of random samples with lower *β*SOR and *β*SIM values than those observed in FC allowed us to obtain *p*‐values and *z*‐scores (alpha set as 0.05). Finally, *γ*‐diversity (total taxonomic richness) was estimated for each catchment and period, and compared considering that RC had more sampling sites than FC (null models were applied like those for *β*‐diversity).

To test our second hypothesis (i.e. replacement of psychrophilic by eurythermic and thermophilic taxa, high temporal turnover and changes in taxonomic composition patterns), changes in water temperature preference between periods (1970s and 2010s) and catchments (FC and RC) were analyzed using LMEs on the relative abundance of psychrophilic, eurythermic and thermophilic taxa. For this, “taxon × temperature preference” (Tachet et al., 2010) and “taxon × site” matrices were crossed to obtain water temperature preference per site for each period. LMEs were analogous to those performed for climatic variables (i.e. considering date and catchment as fixed factors and site as random factor). Nonmetric multidimensional scaling (NMDS) was performed to describe taxonomic composition patterns in the 1970s and 2010s using Bray–Curtis dissimilarity on log‐transformed density data. NMDS was also done separately for summer and winter biological datasets to discard notable differences in species composition patterns between seasons, to be able to focus structural and functional analyses on inter‐decadal changes by using composite samples. Multivariate dispersion was calculated for both basins and periods. Procrustes analysis (least‐squares orthogonal mapping) was used to examine changes in community composition between the 1970s and 2010s for each site. This analysis shows the temporal displacement of each site in multivariate space. Finally, the temporal replacement of taxa was estimated in both catchments using temporal *β*‐diversity (t*β*SOR, Sorensen's dissimilarity) and temporal turnover (t*β*SIM, Simpson's dissimilarity) following Baselga and Orme (2012). The extent to which their compositional divergence between periods was due to different t*β*SOR, t*β*SIM or rates of temporal turnover (t*β*SIM/t*β*SOR; i.e. the rate of temporal dissimilarity explained by the temporal turnover component) between catchments was assessed through one‐way ANOVAs.

#### 2.4.3 Functional changes in invertebrate communities between the 1970s and 2010s

Trait community‐weighted means (TCWM) were calculated to quantify the proportion or weight of each trait modality in each sampled community. Fuzzy correspondence analysis (FCA) was applied on TCWM to explore the functional structure of invertebrate communities (Chevenet et al., 1994). Subsequently, Pearson correlation analysis was performed to look for significant relationships between the first two axes of FCA and functional traits. Five functional indexes widely used to characterize freshwater invertebrate communities (Schmera, Heino, Podani, Erős, & Dolédec, 2017) were also quantified using TCWM for each site and period to account for the changes in functional space at the whole‐community level: FD (Rao's quadratic entropy, Botta‐Dukát, 2005), FRic (Villéger, Mason, & Mouillot, 2008), FEve (Villéger et al., 2008), FDis (Laliberté & Legendre, 2010) and FR (Rosenfeld, 2002). Previously, functional spaces were built (following Maire, Grenouillet, Brosse, & Villéger, 2015) using Gower dissimilarity matrices (adapted for fuzzy‐coded traits, Pavoine, Vallet, Dufour, Gachet, & Daniel, 2009) on either all the functional traits (for the estimation of FRic, FEve and FDis) or only the effect traits (i.e. body size, dispersal, locomotion, food and feeding habits for FD and FR). FR was estimated as the difference between taxonomic diversity (using the Gini–Simpson diversity index) and FD (Pillar et al., 2013). Temporal (between dates) and spatial (between catchments) changes in each functional trait category (using TCWM) and functional indices were analyzed using the “LME procedure” to test hypotheses 3 and 4 (i.e. shifts in taxa functional traits to cope with climate change and decline in functional redundancy and diversity indices, respectively). Given the high number of tests performed for functional traits, Bonferroni correction was applied to *p*‐values.

Homoscedasticity (Levene's test) and normality (Shapiro–Wilk test) of residuals were checked both in LMEs and ANOVAs. All statistical analyses were performed using r statistical software (libraries: “ade4”, “betapart”, “car”, “FD”, “hydroTSM”, “lme4”, “lmerTest”, “multcomp”, “sandwich” and “vegan”; R Development Core Team, 2017). See R code and functions provided in Appendix S2.

## 3 RESULTS

### 3.1 Changes in climatic and flow conditions between the 1970s and 2010s

Climatic variables experienced substantial changes between 1960–1979 and 1996–2015 in both catchments (Table 1, Figure S1.1 in Appendix S1). In particular, mean air temperature (average increase of +0.77°C), maximum air temperature (+2.98°C) and evapotranspiration (+40.07 mm) significantly increased, whereas minimum air temperature (−0.46°C), soil humidity (−0.02), snow‐water equivalence (−17.62 mm) and annual total precipitation (−33.34 mm) significantly decreased in the study area. None of the climatic variables showed different temporal changes between FC and RC (i.e. non‐significant interaction between date and catchment for any of them, Table 1). Mann–Whitney *U* tests pointed that there were no significant differences between years with or without biological samples in terms of total precipitation and air temperature (*p*‐value > 0.05) within each 20 year period (i.e. 1960–1979 and 1996–2015). Complementarily, boxplots showed that biological samples were taken during humid years both in the 1970s and the 2010s (Figure S1.2 in Appendix S1).

**Table 1:** Results of linear mixed‐effect models (LMEs) on climatic variables and water temperature preference of taxa. Marginal *R* ^2^ (*R* ^2^ *m*) and *p‐*values for the whole model and the different terms (date, catchment and the interaction between them) are shown. The sign or trend of the relationship for each term is also displayed that is, temporal (date), spatial (catchment) and spatiotemporal trends (Date: catchment)

	Model		Date		Catchment		Date:Catchment	
	*p*‐value	*R* ^2^ *m*	*p*‐value	Temp. trend	*p*‐value	Trend (greater value)	*p*‐value	Trend (greater value)
Climatic variable								
Mean temperature	**1.09 × 10^−8^**	**0.07**	**1.91 × 10^−8^**	+	0.69	=	0.5	=
Maximum temperature	**4.76 × 10^−14^**	**0.31**	**2.03 × 10^−13^**	+	0.91	=	0.08	=
Minimum temperature	**0.003**	**0.12**	**0.001**	−	0.27	=	0.07	=
Evapotranspiration	**3.84 × 10^−11^**	**0.09**	**1.05 × 10^−10^**	+	0.8	=	0.75	=
Total precipitation	**2.23 × 10^−5^**	**0.44**	**0.012**	−	**0.005**	FC	0.42	=
Solid precipitation	0.22	0.01	0.07	=	0.75	=	0.5	=
Snow‐water equivalence	**0.001**	**0.04**	**0.001**	−	0.83	=	0.32	=
Soil humidity	**6.49 × 10^−6^**	**0.17**	**7.92 × 10^−6^**	−	0.15	=	0.33	=
Water temperature preference of taxa								
Psychrophilic	**7.4 × 10^−4^**	**0.37**	**0.001**	−	0.18	=	0.56	=
Thermophilic	**3.29 × 10^−9^**	**0.74**	**1.95 × 10^−9^**	+	0.56	=	**0.013**	FC (2010s)
Eurythermic	**0.027**	**0.24**	0.11	=	**0.034**	RC	0.1	=

Note

Significant results (if *p*‐value < 0.05) have been highlighted in bold.

FC: free‐flowing catchment; RC: regulated catchment.

There was a generalized decrease in flow magnitude between the 1970s and 2010s in both catchments (Table S1.5). On an average, mean daily flow (MA1) and annual runoff (MA41) are diminished by 14% in RC and FC. Flow reductions were especially strong in summer for both catchments: June (MA17), July (MA18) and August (MA19) flows decreased by 24%, 30% and 22% in RC and by 27%, 6% and 21% in FC, respectively. However, there were differences between catchments since FC flows experienced more and greater changes than RC. Thus, only FC flows decreased in spring and increased in winter: −25% in March (MA14), −34% in April (MA15), −26% in May (MA16), +25% in December (MA23) and +41% in January (MA12). In addition, although annual flow variability (MA42) diminished in both catchments (−9% in RC and −18% in FC), the variability of mean daily and monthly flows decreased in RC (−10% in MA3 and −11% in MA39) but increased in FC (+20% in MA3 and +9% in MA39). In general, these patterns of change in magnitude and variability (based on average flows, i.e. MA) were also observed for maximum (MH) and minimum (ML) flow conditions (Table S1.5 in Appendix S1).

### 3.2 Structural changes in invertebrate communities between the 1970s and 2010s

Across all sites, about 70 and 76 taxa were collected in the 1970s and the 2010s, respectively. In the 1970s, 53 taxa were observed in FC, whereas 62 taxa in RC. In contrast, 65 taxa were recorded in FC, while 56 taxa were collected in RC in the 2010s. Moreover, according to null model results, *γ*‐diversity values of FC did not differ from those estimated for a number of equivalent random sites from RC in the 1970s (*z*‐score = −0.22, *p*‐value > 0.05), but they did in the 2010s (*z*‐score = 4.35; *p*‐value = 0.001; Figure S1.3 in Appendix S1), when FC hosted more taxonomic diversity than RC.

**Figure 2:**
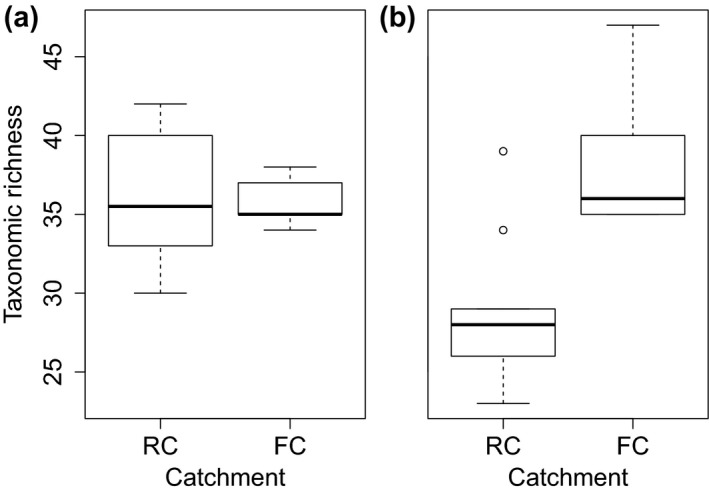
Boxplots showing *α*‐diversity in the free‐flowing (FC) and regulated (RC) catchment in (a) the 1970s and (b) the 2010s. The median is denoted by the bold horizontal line, the box delimits the interquartile range and the whisker lines extend to the observed maxima and minima, except for the outliers symbolized by circles

Although *α*‐diversity did not differ between the 1970s and the 2010s when considering all the stations together (term “Date” in LMEs, *p* = 0.13, *F* = 2.43, *R* ^2^ = 0.11), there were differences between catchments (term “Catchment” in LMEs, *p* = 0.005, *F* = 9.17, *R* ^2^ = 0.43) and a significant interaction between date and catchment (term “Date:Catchment” in LMEs, *p* = 0.004, *F* = 9.92, *R* ^2^ = 0.46). Although both catchments had similar *α*‐diversities in the 1970s (Figure 2), RC experienced a strong reduction in taxonomic richness between the 1970s and the 2010s, whereas it increased in FC.

**Figure 3:**
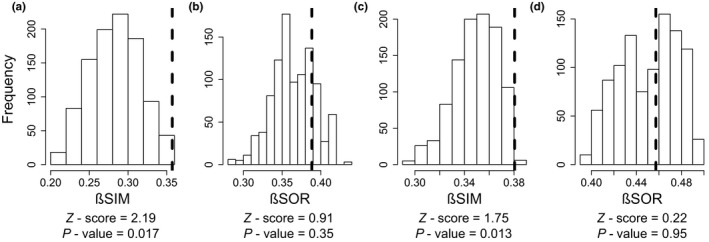
Results of null models comparing spatial turnover (*β*SIM) and *β*‐diversity (*β*SOR) in the 1970s (a and b, respectively) and the 2010s (c and d, respectively) estimated for the free‐flowing catchment (dashed lines that represent empirical values in upper Verdon) and for an equivalent number of randomly selected sites from regulated catchment (histogram values that represent the simulated distributions in upper Durance). *Z*‐scores and *p*‐values are shown for each parameter. *β*SOR: Sorensen's dissimilarity; *β*SIM: Simpson's dissimilarity

Overall spatial *β*‐diversity (*β*SOR) and spatial turnover (*β*SIM) values were similar between the 1970s (*β*SOR = 0.65; *β*SIM = 0.6) and the 2010s (*β*SOR = 0.72; *β*SIM = 0.62). When studying each catchment independently through null models, *β*SOR within FC did not differ from the simulated values estimated for an equivalent number of random localities from RC in the 1970s (null models, *p* = 0.35, *z*‐score = 0.91) nor in the 2010s (*p* = 0.95, *z*‐score = 0.22). On the other hand, *β*SIM in FC was significantly higher than in RC, both in the 1970s (*p* = 0.017, *z*‐score = 2.19) and the 2010s (*p* = 0.013, *z*‐score = 1.75; Figure 3).

**Figure 4:**
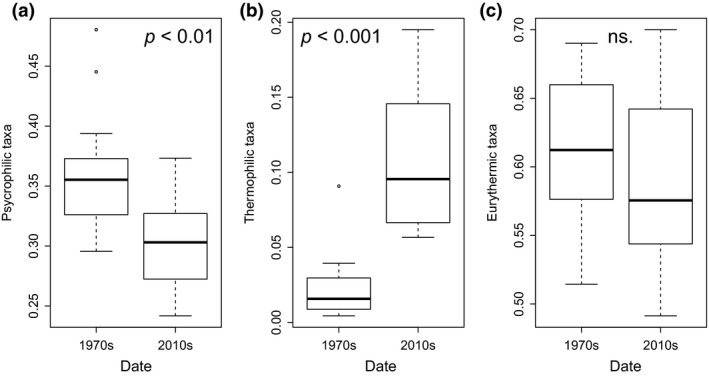
Boxplots showing temporal differences (between the 1970s and the 2010s) in relative abundances of: (a) psychrophilic, (b) thermophilic and (c) eurythermic taxa. The median is denoted by the bold horizontal line, the box delimits the interquartile range, and the whisker lines extend to the observed maxima and minima, except for the outliers symbolized by points. *p*‐values according to linear mixed‐effect models are also provided. Ns.: Non‐significant

The relative abundance of psychrophilic taxa decreased, whereas those of thermophilic taxa increased between the 1970s and the 2010s (Figure 4; Table 1). Psychrophilic taxa decreased similarly in both catchments (LME, term “Date”, *p*‐value = 0.001, *F* = 17.02, *R* ^2 ^= 0.88, Figure S1.4 in Appendix S1), while thermophilic taxa increased more in FC than in RC (LME, interaction “Date:Catchment”, *p* = 0.013, *F* = 7.06, *R* ^2 ^= 0.1; Figure S1.4). There was no change in eurythermic taxa between the 1970s and the 2010s (LME model, *p* > 0.05, Table 1). Psychrophilic taxa *Rhabdiopteryx neglecta *and *Amphinemura *spp. (Plecoptera) were initially (1970s) abundant in all sampling sites but disappeared in more than 50% and 40% of the sites of the study area, respectively (with a reduction in density of: −99.9% and −65.4%, respectively). Similarly, other psychrophilic taxa such as *Dictyogenus alpinus *(Plecoptera) and *Drusus discolor* (Trichoptera), occurring in more than 30% of all sampling sites in the 1970s, were only observed at one site in the 2010s (with strong reductions in density of −98.6% and −62.7%, respectively). Thermophilic and eurythermic taxa such as *Serratella ignita*, *Habroleptoides* spp. and *Oligoneuriella rhenana* (Ephemeroptera) were absent in the 1970s but have since colonized FC (appearing in 80%, 60% and 40% of the FC sites in the 2010s, respectively). Other tolerant Ephemeroptera as *Acentrella sinaica* or *Caenis *spp. which were marginally present (two sites) in the 1970s doubled their occurrences and greatly increased in abundance (+6,522% and +6,404% respectively) in the 2010s, especially in FC.

**Figure 5:**
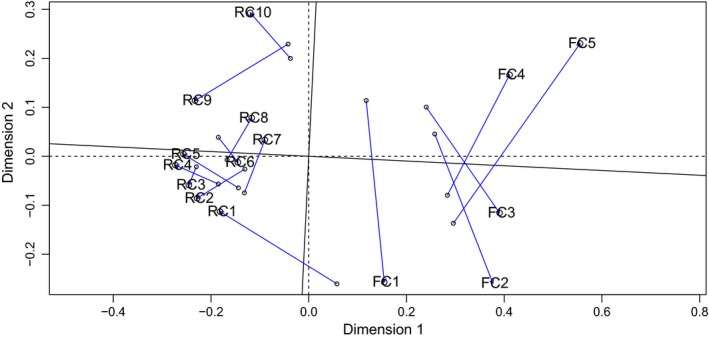
Nonmetric multidimensional scaling‐Procrustes plot comparing the compositional patterns for regulated (RC) and free‐flowing (FC) catchments between periods. Note that blue lines represent the displacement of each site within the multivariate space between the 1970s (points) and the 2010s (label) [Colour figure can be viewed at http://www.wileyonlinelibrary.com/]

Community composition patterns were more similar between FC and RC in the 1970s (with more shared taxa between them) than in the 2010s (Figure 5). RC experienced a homogenization in terms of composition with a reduction in multivariate dispersion, whereas FC showed a slight increase in multivariate dispersion and some compositional diversification (Figure S1.5 in Appendix S1). The same composition patterns were observed when considering summer and winter samples independently (Figure S1.6 in Appendix S1).

**Figure 6:**
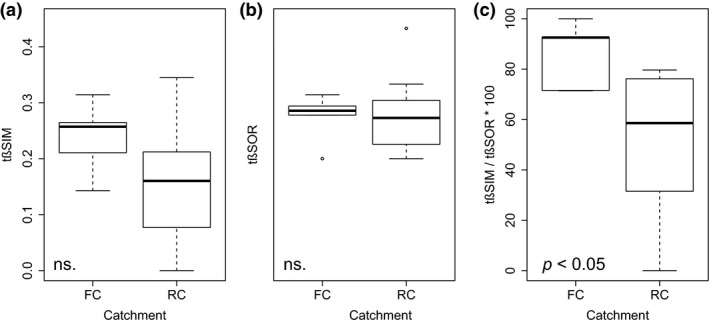
Boxplots showing t*β*SOR components: (a) t*β*SIM, b) t*β*SIM and c) t*β*SIM/t*β*SOR in free‐flowing (FC) and regulated (RC) catchments. Significant changes according to ANOVAs are shown. The median is denoted by the bold horizontal line, the box delimits the interquartile range, and the whisker lines extend to the observed maxima and minima, except for the outliers symbolized by points. Ns.: non‐significant.; t*β*SOR: Sorensen's dissimilarity; t*β*SIM: Simpson's dissimilarity

The temporal variation (t*β*SOR, mean and median = 0.28, *SD* = 0.06) of taxonomic assemblages between the 1970s and the 2010s was dominated by taxa turnover (t*β*SIM, mean and median = 0.17, *SD* = 0.1). Turnover represented, on average, 60% of the total compositional changes across the whole study area (t*β*SIM/t*β*SOR, mean = 0.61, median = 0.72, *SD* = 0.31). When considering each catchment independently, significant differences were found between them (ANOVA, *p* = 0.027, *F* = 6.24, *R* ^2^ = 0.32; Figure 6). Higher rates of temporal turnover (t*β*SIM/t*β*SOR) were found in FC (mean = 0.86, median = 0.92, *SD* = 0.13) in comparison to RC (mean = 0.49, median = 0.58, *SD* = 0.31).

### 3.3 Functional changes in invertebrate communities between the 1970s and 2010s

**Figure 7:**
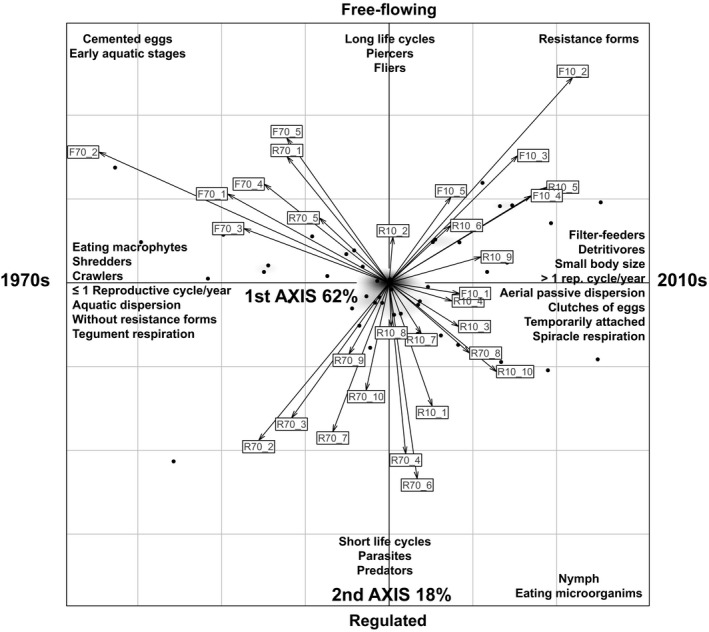
Ordination plot showing the distribution of samples (boxes) and the most significant functional traits (*R* > 0.4 and *p* < 0.05 in fuzzy correspondence analysis [FCA] correlations) along the two first axes of FCA (80% of explained variance). Note that samples taken from the free‐flowing and regulated catchments are preceded by F and R, respectively, followed by the collection date (70 for the 1970s and 10 for the 2010s) and the code of the sampling site

Fuzzy correspondence analysis revealed temporal (1970s/2010s samples distributed from left to right along the first axis which explained 62% of the total variability) and spatial (FC/RC distributed along the second axis which explained 18%) effects on the functional structure of aquatic invertebrate communities (Figure 7). Both axes were significantly correlated (*R* > 0.38; *p < *0.05) with a wide range of functional traits (Figure 7, Table S1.6 in Appendix S1). Similarly, LMEs detected a severe shift in functional traits related to life cycle, physiology, behavior, resistance and resilience between the 1970s and the 2010s in both catchments (Table 2; see detailed results in Table S1.7 in Appendix S1). With the exception of life cycle duration, all functional traits experienced changes between both periods, some of them being more intense in FC than in RC (LME, interaction term “Date:Catchment” significant for body size, food preference and reproduction, Table 2). Regarding life cycle, the main temporal changes included reductions in body size and in the abundance of taxa with aquatic larva and eggs, as well as the increment of multivoltinism (>1 reproductive cycle per year). In relation to resistance and resilience traits, reduction of crawlers, burrowers and taxa with aquatic dispersal was observed. On the other hand, increases in taxa with aerial dispersal and temporary attachment were detected. A proliferation of taxa with resistance forms (eggs or staboblasts and diapause or dormancy) to the detriment of those without resistance forms was also observed. With respect to behavioral traits, taxa reproducing by egg clutches increased, whereas those using cemented, isolated eggs decreased. The main temporal changes in food preference and feeding habits were characterized by the spread of filter feeders, detritivores and taxa eating microinvertebrates, and the reduction of shredders and taxa eating macrophytes. Regarding respiration strategies, taxa using tegument decreased while those having plastron and spiracle increased. Finally, when comparing free‐flowing and regulated catchments, more fliers, piercers and taxa with earlier aquatic stages and reproduced by cemented eggs were detected in FC in comparison with RC (in both periods).

**Table 2:** Results of linear mixed‐effect models (LMEs) on biological traits of aquatic invertebrates. Only significant results after applying Bonferroni correction are displayed (i.e. *p*‐value < 0.001; complete results can be found in Table S1.7 in Appendix S1). Marginal *R* ^2^ (*R* ^2^ *m*) and *p‐*values for the whole model and the different terms (date, catchment and the interaction between them) are shown. The signs or trends of the relationships are also displayed, i.e. temporal (date), spatial (catchment) and spatiotemporal trends (Date:Catchment)

Biological trait	Categories	Code	Model		Date		Catchment		Date:Catchment	
			*p*‐value	*R* ^2^ *m*	*p*‐value	Temporal trend (1970s−2010s)	*p*‐value	Spatial trend (greater value)	*p*‐value	Spatio‐temporal trend (greater value)
Body size	>0.25 −0.5cm	T1_2	4.89 × 10^−7^	0.63	2.08 × 10^−7^	+	0.82	=	0.002	=
	>1–2cm	T1_4	3.99 × 10^−10^	0.77	1.95 × 10^−9^	−	0.001	=	5.85 × 10^−5^	FC (1970s)
Potential number of cycles per year	<1	T3_1	3.5 × 10^−4^	0.43	0.001	−	0.032	=	0.28	=
	1	T3_2	4.38 × 10^−4^	0.42	5.45 × 10^−4^	−	0.068	=	0.011	=
	>1	T3_3	1.13 × 10^−5^	0.55	2.6 × 10^−5^	+	0.012	=	0.006	=
Aquatic stages	Egg	T4_1	1.82 × 10^−5^	0.53	1.6 × 10^−4^	−	0.005	=	0.002	=
	Larva	T4_2	1.83 × 10^−5^	0.53	8.6 × 10^−4^	−	8 × 10^−4^	FC	0.003	=
	Nymph	T4_3	1.21 × 10^−5^	0.55	2.35 × 10^−4^	+	0.002	=	0.002	=
Reproduction	Isolated eggs, free	T5_2	1.28 × 10^−7^	0.66	5.31 × 10^−7^	+	0.001	=	0.069	=
	Isolated eggs, cemented	T5_3	1.47 × 10^−8^	0.71	1.09 × 10^−7^	−	7.05 × 10^−4^	FC	3.34 × 10^−4^	FC (1970s)
	Clutches, cemented or fixed	T5_4	2.09 × 10^−7^	0.65	2.31 × 10^−6^	+	2.6 × 10^−4^	RC	0.017	=
Dispersal	Aquatic active	T6_2	7.28 × 10^−6^	0.56	2.86 × 10^−6^	−	0.42	=	0.007	=
	Aerial passive	T6_3·	3.72 × 10^−8^	0.69	1.72 × 10^−8^	+	0.99	=	0.003	=
Locomotion and substrate relationship	Flier	T21_1	2.54 × 10^−10^	0.78	8.8 × 10^−6^	+	7.15 × 10^−6^	FC	0.017	=
	Crawler	T21_4	1.87 × 10^−5^	0.53	1.03 × 10^−5^	−	0.24	=	0.004	=
Locomotion and substrate relationship	Burrower	T21_5	6.27 × 10^−4^	0.41	7.66 × 10^−4^	−	0.15	=	0.63	=
	Temporarily attached	T21_7	6.26 × 10^−9^	0.72	3.32 × 10^−9^	+	0.95	=	0.004	=
Resistance forms	Eggs, statoblasts	T7_1	9.71 × 10^−5^	0.48	9.71 × 10^−4^	+	0.08	=	0.98	=
	Diapause or dormancy	T7_4	6.73 × 10^−8^	0.68	4.52 × 10^−8^	+	0.11	=	0.07	=
	No resistance forms	T7_5	6.05 × 10^−7^	0.61	1.11 × 10^−6^	−	0.024	=	0.1	=
Food preference	Detritus < 1mm	T8_2	3.21 × 10^−6^	0.58	4.98 × 10^−5^	+	0.028	=	0.3	=
	Living macrophytes	T8_5	1.37 × 10^−8^	0.72	1.09 × 10^−8^	−	0.29	=	6.5 × 10^−4^	FC (1970s)
	Living microinvertebrates	T8_7	3.72 × 10^−6^	0.58	4.3 × 10^−6^	+	0.19	=	4.8 × 10^−4^	FC (2010s)
Feeding habits	Shredder	T9_3	5.12 × 10^−7^	0.63	3.82 × 10^−7^	−	0.16	=	0.001	=
	Filter‐feeder	T9_5	1.09 × 10^−8^	0.71	5.45 × 10^−9^	+	0.99	=	0.004	=
Respiration	Tegument	T10_1	1 × 10^−6^	0.61	3.81 × 10^−7^	−	0.45	=	0.1	=
	Plastron^a^	T10_3	1.27 × 10^−11^	0.82	6.21 × 10^−6^	+	7.1 × 10^−7^	FC	0.01	=
	Spiracle	T10_4	5.28 × 10^−9^	0.73	4.22 × 10^−9^	+	0.15	=	0.06	=

Note

FC: free‐flowing catchment; RC: regulated catchment.

a Results that despite being statistically significant did not meet model assumptions after transformation.

**Figure 8:**
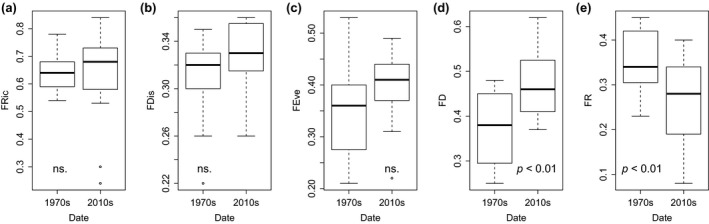
Boxplots showing the temporal differences in functional indexes: (a) functional richness (FRic), (b) functional dispersion (FDis), (c) functional evenness (FEve), (d) functional diversity (FD) and (e) functional redundancy (FR) between the 1970s and the 2010s. The median is denoted by the bold horizontal line, the box delimits the interquartile range, and the whisker lines extend to the observed maxima and minima, except for the outliers symbolized by points. Significant changes according to linear mixed‐effect models (*p* < 0.05) are shown. Ns.: Non‐significant

Only FD (LME model, *p*‐value = 0.009, *R* ^2^ *m* = 0.28) and FR (*p*‐value = 0.013, *R* ^2^ *m* = 0.25) showed significant changes through time or across space. In particular, FD increased from the 1970s to the 2010s (LME, term “Date”, *p*‐value = 0.007, *F* = 10.2, *R* ^2^ = 0.91), whereas FR decreased in the same period (term “Date”, *p*‐value = 0.004, *F* = 10.14; *R* ^2^ = 0.94, Figure 8). None of them showed significant spatial differences between catchments or interactions between date and catchment. FRic (LME model, *p*‐value = 0.061), FEve (*p*‐value = 0.3) and FDis (*p*‐value = 0.165) did not change through time or across catchments (terms “Date”, “Catchment” and the interaction between them showed *p*‐value > 0.05).

## 4 DISCUSSION

This study yields new insights into the response of stream invertebrate communities to climate change in alpine catchments, in interaction with preexisting anthropogenic stressors such as flow regulation. As expected, climate turned warmer and drier between the 1970s and the 2010s in the study area. Cold‐adapted taxa have been partially replaced by warming‐tolerant ones, resulting in high spatial and temporal turnover, as well as changes in taxonomic and functional structure and diversity. The spread of taxa with functional strategies different to those previously present (e.g. increase of multivoltine taxa with small body size, resistance forms, reproducing mainly by clutches and dispersing by air), combined with the tolerance of some resident species to present‐day conditions has resulted in an unexpected increase of FD. Nevertheless, a reduction in FR was detected, which could reduce the ability of aquatic communities facing an intensification of ongoing climate change or new anthropogenic disturbances (i.e. reduced resistance and resilience). Taxonomic responses differed between free‐flowing and regulated catchments suggesting synergistic effects between climate change and flow regulation on taxonomic structure and diversity. However, these taxonomic changes did not translate into different functional adaptations of taxa to climate change between both types of catchments. Thus, a progressive replacement of a specialized aquatic community by a more ubiquitous and generalist one seems to be occurring in both free‐flowing and regulated catchments.

### 4.1 Changes in climatic and flow conditions between the 1970s and 2010s

Climate change affected both alpine catchments. One of the main shifts was a noteworthy rise of mean daily maximum temperature (+3°C) between both periods. The slight decrease in mean daily minimum temperature (−0.46°C) could have buffered the increase in mean daily temperature (+0.77°C), which may seem relatively low compared to the range reported by the fifth IPCC report (1–2°C between 1970 and 2004 in the area; IPCC, 2013). Air temperature is one of the most important predictors of invertebrate composition in climatically restricted ecosystems (Mustonen et al., 2018) since increases in air temperature are generally accompanied by rising water temperatures (Caissie, 2006; Livingstone & Lotter, 1998; Mohseni, Erickson, & Stefan, 1999). Such temperature changes were associated with changes in evapotranspiration, annual precipitation and snow water equivalence, indicating a shift towards warmer and drier climatic conditions.

General reductions in flow magnitude and interannual variability were also observed. The substantial reductions observed in mean annual flows (−14%) were similar than those predicted for the nearby Rhône River (in which both catchments flow) during the last few decades of the 21st century (>−10%; Huss & Hock, 2018; van Vliet et al., 2013). In concordance with predictive hydrological models (Fatichi, Rimkus, Burlando, Bordoy, & Molnar, 2013), decreases in summer flows were even far greater, showing a consistent reduction across gauging stations (reaching values of −30%). In addition, spring monthly flows also decreased notably in FC where no glacier influence was present (reaching values of −34%), which might be linked to advancing snowmelt season (Barnett, Adam, & Lettenmaier, 2005). Decreases in discharge usually cause reductions in water velocity, water depth and wetted channel width, while increasing sedimentation, changing thermal regime and water chemistry (Dewson et al., 2007; McGregor et al., 1995). Despite the dominant natural land use of the study area, the construction of water treatment plants in recent decades and conservation efforts, these hydro‐climatic changes can reduce pollution sensitive taxa and increase tolerant ones (Floury, Usseglio‐Polatera, Ferreol, Delattre, & Souchon, 2013). Flow reductions may also exacerbate the increase of water temperatures in summer (van Vliet, Ludwig, Zwolsman, Weedon, & Kabat, 2011). On the basis of anticipated shrinkage of snowpack and reductions of glacier extent, the volume of snowmelt would continue decreasing in the future. In addition, the timing of peak snow‐ and ice‐melt would shift to winter and early spring (Barnett et al., 2005; Hannah et al., 2007), which could lead to even stronger late spring and summer flow reductions in alpine rivers.

### 4.2 Structural and functional responses of aquatic invertebrate communities

Changes in taxonomic composition, structure and functioning of invertebrate communities have been detected between the 1970s and the 2010s in our study area. Climate change reduces the habitat of psychrophilic species, especially in alpine ecosystems (Hotaling et al., 2017; Vittoz et al., 2013). In particular, rivers are experiencing northward range shifts of warm‐water species while some cold‐water species may go locally extinct, particularly in high altitudinal and latitudinal areas, where species distributions are most obviously limited by elevation and climate (Domisch et al., 2013; Heino, Virkkala, & Toivonen, 2009; Mustonen et al., 2018). Thus, low water temperature, high solute concentrations and varying hydrological regimes have restricted the fauna to a few specialized species in alpine rivers. When aquatic species exhibit high dispersal abilities, warming and more stable hydrological regimes enhance species diversity due to the spread of thermophilic and opportunistic species to the detriment of psycrophilic ones (Hotaling et al., 2017; Jacobsen et al., 2012). However, hydrological alterations could limit colonization and dispersal of thermophilic species able to establish under the new climatic conditions in regulated rivers, due to habitat homogenization (Belmar, Bruno, Martínez‐Capel, Barquín, & Velasco, 2013; Heino et al., 2009) and disruption of longitudinal connectivity associated with hydropower production (Ward & Stanford, 1983). Consequently, our results (hypothesis 1) showed that temporal patterns of *α*‐ and *γ*‐ diversities diverged between catchments, with increases in the free‐flowing (as also found by Brown et al., 2007) but decreases in the regulated one associated with biotic homogenization. Similarly, although both catchments exhibited high temporal (hypothesis 2) and spatial turnover (*β*‐diversity), these patterns were stronger in the free‐flowing one. The absence of hydroelectric infrastructures enables greater spatial environmental variability, availability and diversity of mesohabitats (Rahel, 2002), and ecological integrity (Bunn & Arthington, 2002), promoting higher colonization rates in free‐flowing catchments. In a context of climate change, a more intense replacement of taxa over time and space in free‐flowing versus regulated rivers could allow the establishment of more heterogeneous, diverse and resilient communities. However, this pattern may be temporary since some of the colonizing species might outcompete the native ones as climate change intensifies in alpine rivers (Hotaling et al., 2017; Vittoz et al., 2013).

The observed increases in taxonomic diversity were related to decreases in abundance and occurrence of some taxa with particular combinations of traits (large body size, aquatic dispersion, low reproduction rates, aquatic sensitive stages and lack of resistance forms; hypothesis 3) but without disappearing completely. Meanwhile, taxa showing suitable functional traits (small body size, multivoltinism, aerial dispersion, resistance forms and specialized respiration techniques as plastron or spiracle) to survive under the new climatic conditions (flow reduction and temperature rising), are more likely to colonize new habitats and expand their distributional range, especially in free‐flowing rivers. According to the functional trends detected in our study area (alpine catchments flowing to the Mediterranean Sea), invertebrate communities are becoming more similar to those typical of Mediterranean rivers (Bonada et al., 2007), which could ultimately affect primary and secondary production, biogeochemical processes and nutrient cycles (Wilby, 2008).

The observed pattern in functional traits is closely related to the increase of FD and the decrease of FR (hypothesis 4). Contrary to expected trends in more temperate areas, colonization of climatically restricted ecosystems by functionally different taxa may produce an increase in FD in the context of climate change (Brown et al., 2018). FRic, FEve and FDis (estimated using all traits) followed a similar, but not significant, pattern than FD (estimated only with effect traits), which could be due to a greater influence of climate change on effect traits (those used for FR and FD estimation). Accordingly, our results showed that the arrival and expansion of taxa with new effect traits (i.e. small body size and aerial dispersion) increased FD while local extinctions reduced FR due to the loss of taxa with effect traits that were no longer frequent (i.e. large body size and aquatic dispersion). Therefore, aquatic invertebrate communities seem to be in the middle of a climate‐driven transition phase characterized by the progressive colonization and spread of taxa with more suitable traits to survive under the new environmental conditions and the gradual loss of cold‐adapted ones (which showed a different trait combination). This biological transition entails a temporarily increase in FD (now there is a greater variety of effect traits in the study area) but a decrease in FR (less taxa sharing the same effect trait combination). Functional redundancy, which represents the number of species contributing similarly to an ecosystem function, relates positively to the ecosystem stability, resistance and resilience (Hooper et al., 2005). Thus, the loss of individuals and species contributing to the same ecosystem functions increases the risk of ecosystem failure related to climate change intensification or other anthropogenic disturbances (Bruno, Gutierrez‐Cánovas, Sanchez‐Fernández, Velasco, & Nilsson, 2016). In the future, as climate change and the subsequent replacement of species intensify, as initially hypothesized, a decrease of FD could be observed. Finally, although observed effects of climate change on aquatic invertebrates are clear and supported by robust outcomes, sampling effect could have exerted certain influence given the limited dataset and temporal replication. Therefore, the biological responses to climate change and flow regulation found here should be followed by empirical research incorporating additional information such as hydropeaking frequency and magnitude, environmental flows and additional invertebrate samples in order to reach more extensive conclusions and improve river management.

### 4.3 Management implications

Climate change can negatively affect dam management since reduced flows jeopardize the ability of alpine rivers to satisfy current water uses (van Vliet et al., 2016). Reservoirs fed with water coming from glaciers and snowpacks will not be able to maintain the same water levels in summer and autumn, when demand is greatest (Barnett et al., 2005). In fact, a transition from ice‐ and snow‐fed to more rain‐fed rivers has been recently detected as a consequence of climate change in the region (Van Vliet et al., 2013). Furthermore, decreases in snow cover between −24% and −55% (depending on the climatic scenarios) are expected for the second half of this century in the study area, which will be coupled with a decrease in total runoff (Etchevers, Golaz, Habets, & Noilhan, 2002). In this climatic context, reduction in snow‐ and ice cover can enhance sediment transport and deposition in alpine rivers with strong effects on water quality and quantity, aquatic habitat, flooding, infilling of hydropower reservoirs, turbine abrasion and agricultural and infrastructural development (Schaefli, Hingray, & Musy, 2007). Thus, under climate change, a major challenge is to satisfy multiple water uses as hydropower production or water abstraction for irrigation, while preserving biodiversity, ecological functions and associated ecosystem services (Arthington, Naiman, Mcclain, & Nilsson, 2010).

The survival of many species under climate change will depend on their ability to disperse and colonize new favorable sites (Root, Price, Hall, & Schneider, 2003), but could be limited by the recurrent fragmentation along alpine river networks. The cumulative effect of flow regulation entails a reduced discharge and the disruption of longitudinal connectivity, which may imply a greater isolation of alpine aquatic communities and reduced resilience (Maiolini & Bruno, 2007). Thus, free‐flowing river communities can be more resilient to climate change than regulated ones, for which proactive and adaptive environmental flow management, as well as restoration measures at catchment scale, seem necessary to maintain and increase longitudinal and lateral connectivity, which are essential to the viability of populations of many aquatic species (Arthington et al., 2010; Bunn & Arthington, 2002; Palmer et al., 2008).
