# Supplementary material for: Structural and functional responses of invertebrate communities to climate change and flow regulation in alpine catchments
Source: Glob Chang Biol. 2019 Mar 3;25(5):1612–28. doi: 10.1111/gcb.14581 (PMC6850064; doi:10.1111/gcb.14581)

Appendix S1. Supplementary tables and figures explaining environmental and biological variables and patterns.

Table S1.1. Geographic information of the sampling sites.

Sampling sites	Abreviation	Altitude	Catchment	X	Y	Data code
Plampinet	PLA	1600	Upper Durance (RC1)	941506	2007656	DUR1
Fontenil	FON	1330	Upper Durance (RC2)	941298	1998543	DUR2
Briançon	BRI	1180	Upper Durance (RC3)	937221	1994432	DUR3
Prelles	PRE	1130	Upper Durance (RC4)	935588	1992048	DUR4
L'argentiére	ARG	975	Upper Durance (RC5)	934326	1987364	DUR5
Roche de Rame	ROC	940	Upper Durance (RC6)	935077	1982347	DUR6
St Clément	STC	870	Upper Durance (RC7)	936384	1968659	DUR7
Embrun	EMB	800	Upper Durance (RC8)	931832	1960000	DUR8
Maison du Roy (Guil)	GUT	1200	Upper Durance (RC9)	949503	1977915	GU1
Simoust (Guil)	GUL	895	Upper Durance (RC10)	940895	1972611	GU2
Les Gays	GAY	1620	Upper Verdon (FC1)	938754	1928102	VER2
Allos	ALL	1415	Upper Verdon (FC2)	942314	1925086	VER3
Colmars	COL	1260	Upper Verdon (FC3)	943685	1919166	VER4
Thorame	THO	1030	Upper Verdon (FC4)	939563	1904089	VER5
Saint André les Alpes	STA	900	Upper Verdon (FC5)	935462	1893410	VER6

RC = Regulated catchment; FC = Free-flowing catchment. Geographical coordinates (X and Y) are provided according to the Datum RGF93 and the projection Lambert-93.

Table S1.2. Mean densities of the taxa identified for the 1970s and the 2010s at the different sites. See Table S1.1 for the description of the sites.

	1970s															2010s														
TAXON/SITE	DU1	DU2	DU3	DU4	DU5	DU6	DU7	DU8	GU1	GU2	VE2	VE3	VE4	VE5	VE6	DU1	DU2	DU3	DU4	DU5	DU6	DU7	DU8	GU1	GU2	VE2	VE3	VE4	VE5	VE6
Acentrella sinaica	0	0	0	0	0	0	0	0	0	0	0	0	0	1	8	0	0	0	0	0	0	0	0	0	0	0	2	4	373	202
Agraylea	0	0	0	0	0	0	0	0	0	0	0	0	0	0	0	0	0	0	0	0	0	0	0	0	29	2	0	0	0	0
Allogamus auricollis	9	6839	2968	128	572	320	2329	720	3	48	0	0	0	0	0	1265	402	1103	177	199	1234	456	2214	5429	119	4	0	0	7	0
Allotrichia	0	0	0	0	0	0	0	0	0	0	0	0	0	0	0	0	0	1	0	0	0	1	4	60	70	4	0	0	0	0
Amphinemura	286	83	3	7	27	1	42	4	2	3	4	94	5	185	50	2	3	3	0	0	0	0	1	0	0	45	67	14	118	24
Anthomyidae	0	0	0	1	0	0	0	0	1	0	0	0	3	0	0	0	0	0	0	0	0	0	0	1	0	0	0	0	0	0
Atherix	77	0	0	1	1	0	4	0	13	5	9	25	19	38	28	0	0	0	3	0	0	0	0	0	5	2	29	107	35	0
Baetis alpinus	2824	1462	939	835	2730	1645	948	658	1857	1105	579	260	796	1031	309	952	2400	819	3417	1935	2014	1786	1602	1837	3520	286	144	762	473	83
Baetis buceratus	0	0	0	0	0	0	0	0	0	0	0	0	0	0	0	0	0	0	0	0	0	0	0	0	0	0	0	0	0	4
Baetis fuscatus	0	0	0	0	0	0	0	2	0	0	0	0	0	0	0	0	0	0	0	0	0	0	0	0	0	0	0	0	0	3
Baetis lutheri	0	0	0	0	0	0	0	0	0	0	0	0	0	0	0	0	0	0	0	0	0	0	0	0	0	0	0	0	22	11
Baetis melanonyx	7	1	1	3	8	99	2	1	369	541	45	18	20	0	0	362	35	36	6	1	19	8	8	190	99	242	5	14	0	0
Baetis muticus	1	0	2	8	36	0	23	2	1	0	66	58	16	33	11	3	0	0	0	1	0	1	0	2	6	27	24	23	29	14
Baetis rhodani	3	39	3	1	3	1	16	39	1	10	43	33	39	69	30	0	2	0	3	9	23	83	210	4	376	2	5	26	374	101
Baetis scambus	0	0	0	0	0	0	0	0	0	0	0	0	0	0	0	0	0	0	0	0	0	0	0	0	0	0	0	4	0	0
Baetis vernus	0	0	0	0	2	0	2	10	0	1	0	0	0	35	0	0	0	0	0	0	0	6	0	0	1	62	187	303	83	2
Blephariceridae	261	1	1	1	0	0	0	0	5	26	0	0	0	0	0	29	4	0	0	0	9	33	6	4	1	2	0	0	0	0
Brachyptera	0	0	0	0	0	0	0	0	0	0	0	0	0	0	0	0	0	0	0	0	0	0	0	0	0	0	0	0	0	3
Bythinella	0	0	0	0	0	0	0	0	0	0	0	0	0	0	0	0	0	0	0	0	0	0	0	0	1	0	0	0	0	0
Caenis	0	0	0	0	0	0	0	1	0	0	0	0	0	1	0	0	0	0	0	0	0	0	0	0	0	0	2	9	60	65
Capnia	505	47	155	190	100	174	26	18	0	0	13	3	6	0	1	5	0	0	3	0	0	0	0	0	0	0	2	0	8	29
Capnioneura	0	0	0	0	0	0	0	0	0	0	0	0	0	0	0	0	0	0	0	0	0	0	0	0	0	13	3	0	2	3
Ceratopogonidae	9	1	1	1	0	0	0	0	0	2	0	1	0	0	0	4	30	0	1	0	0	7	0	5	5	0	0	0	3	0
Chironomidae	0	0	0	0	0	0	0	0	0	0	0	404	2817	529	595	0	0	0	0	0	0	0	0	0	0	0	0	0	0	0
Chironomini	0	4	2	7	4	8	122	98	0	0	0	4	5	0	11	0	0	0	0	0	0	0	0	1	7	0	0	3	9	19

	1970s															2010s														
TAXON/SITE	DU1	DU2	DU3	DU4	DU5	DU6	DU7	DU8	GU1	GU2	VE2	VE3	VE4	VE5	VE6	DU1	DU2	DU3	DU4	DU5	DU6	DU7	DU8	GU1	GU2	VE2	VE3	VE4	VE5	VE6
Chloroperla	49	0	0	0	0	0	1	0	0	0	0	0	0	3	0	0	0	0	0	0	0	0	0	0	0	0	0	2	0	8
Chrysopilus	0	0	0	0	0	0	0	0	0	0	0	0	0	0	0	0	0	0	0	0	0	0	0	0	3	0	0	0	0	0
Crenobia Alpina	365	37	22	78	33	2	3	0	8	11	24	3	0	0	0	10	69	51	14	13	12	0	2	41	3	6	13	3	2	0
Cryptothrix	24	0	0	0	0	0	0	0	0	0	0	0	0	0	0	0	0	0	0	0	0	0	0	0	0	0	0	0	0	0
Dictyogenus	46	0	0	1	1	0	0	0	0	0	16	5	0	0	35	0	0	0	0	0	0	0	0	0	0	2	0	0	0	0
Dinocras	20	13	2	5	77	4	83	25	0	0	0	0	0	3	139	0	0	0	0	0	0	0	0	0	0	0	0	0	5	18
Dixa	1	0	0	1	0	0	0	0	0	0	0	0	0	0	0	0	0	0	0	0	0	0	0	0	1	0	0	0	0	0
Drusus	14	0	0	0	0	0	0	0	0	1	5	8	5	1	0	0	0	0	0	0	0	0	0	0	0	13	0	0	0	0
Drusus discolor	2	0	0	0	0	0	0	0	0	1	0	0	0	0	0	0	0	0	0	0	0	0	0	0	0	2	0	0	0	0
Dytiscidae	0	0	0	0	0	0	1	0	0	0	0	0	0	0	0	0	0	0	0	0	0	0	0	0	0	0	0	0	0	0
Ecdyonurus	309	53	23	28	58	42	161	10	8	8	10	10	14	19	18	14	12	9	1	5	18	24	36	20	101	13	20	32	20	23
Elmis	0	0	0	0	0	1	1	1	0	0	0	0	0	1	8	0	0	0	0	0	0	0	0	0	0	0	0	0	7	0
Empididae	41	30	7	4	7	13	6	33	18	34	8	25	18	55	35	38	28	47	7	30	11	7	10	39	36	38	5	5	9	27
Epeorus	15	0	2	5	0	1	0	0	3	1	13	24	0	11	1	3	0	4	3	1	2	2	4	0	0	4	4	0	2	16
Ephemerella	0	0	0	0	0	0	0	0	0	0	0	0	0	0	0	0	0	0	0	0	0	0	0	0	0	0	2	23	25	9
Ephydridae	0	0	3	0	0	0	0	0	0	0	0	0	0	0	0	0	0	0	0	0	0	0	0	0	0	0	0	0	0	0
Esolus	0	0	1	0	0	0	0	0	0	0	5	65	6	0	0	0	0	0	0	0	0	0	1	0	1	3	4	0	6	5
Gammarus	0	9	3	1	2	1	20	13	0	0	0	0	0	0	0	0	0	3	0	10	2	0	4	9	7	0	0	0	2	0
Glossosoma	8	0	0	0	0	0	14	0	0	0	1	3	2	3	0	0	0	1	0	0	0	12	11	0	4	5	0	0	0	0
Habroleptoides	0	0	0	0	0	0	0	0	0	0	0	0	0	0	0	0	0	0	0	0	0	0	0	0	0	9	63	8	0	0
Habrophlebia	0	0	0	0	0	0	0	0	0	0	3	3	0	0	0	0	0	0	0	0	0	0	0	0	0	0	0	0	0	0
Hydra	0	0	0	1	4	0	2	1	0	0	0	0	0	0	0	0	0	0	0	0	0	0	0	0	0	0	0	0	0	0
Hydraena	5	1	2	2	1	3	4	0	0	0	0	8	3	4	1	3	2	3	1	0	0	1	0	1	0	10	5	5	2	0
Hydropsyche	0	0	0	0	2	0	0	1	0	0	0	0	0	31	34	0	0	0	0	1	1	20	31	141	362	13	17	142	282	23
Hydroptila	0	0	0	0	0	0	0	0	1	0	0	0	0	0	0	0	0	0	0	0	0	0	1	0	3	0	0	0	0	0
Isoperla	225	14	0	11	16	10	27	3	5	1	19	0	1	201	46	19	7	1	0	0	5	4	11	1	26	4	2	16	64	12
Leptophlebiidae	0	0	0	0	0	0	0	0	0	0	0	0	3	0	0	0	0	0	0	0	0	0	0	0	0	0	0	0	0	0
Leuctra	5926	2598	2272	8008	8246	393	1918	892	2079	1095	784	1358	783	2705	1494	1156	2938	723	1658	1096	1290	726	925	1403	358	680	201	527	682	308

	1970s															2010s														
TAXON/SITE	DU1	DU2	DU3	DU4	DU5	DU6	DU7	DU8	GU1	GU2	VE2	VE3	VE4	VE5	VE6	DU1	DU2	DU3	DU4	DU5	DU6	DU7	DU8	GU1	GU2	VE2	VE3	VE4	VE5	VE6
Limnephilidae	77	1	0	0	0	0	0	1	0	0	4	4	0	55	0	0	0	0	0	0	0	0	0	2	0	3	0	0	0	0
Limnius	0	0	0	0	0	0	0	0	0	0	0	0	0	0	1	0	0	0	0	0	0	0	0	0	0	0	0	0	39	2
Limoniidae	116	63	25	34	49	26	19	13	38	96	41	103	75	33	14	109	140	56	39	45	67	40	29	92	30	38	32	67	59	23
Melampophylax mucoreus	0	0	0	0	0	0	0	0	0	0	0	0	0	0	0	0	0	0	0	0	0	0	0	0	0	0	0	0	2	0
Nemoura	110	2	99	49	48	11	13	6	0	0	47	13	27	4	3	0	0	1	1	0	3	1	0	0	0	9	2	2	2	4
Nemoura mortoni	115	23	21	14	13	3	40	5	5	9	314	53	0	0	0	0	0	0	0	0	0	2	0	4	2	2	10	0	0	0
Odontocerum albicorne	0	0	0	0	0	0	0	0	0	0	0	0	0	0	0	0	0	0	0	0	0	0	0	0	0	0	0	0	3	0
Oligochaeta	109	223	25169	4428	477	2644	2153	395	18	13	686	28	30	8	19	7	21	59	207	45	56	34	27	94	1287	4	2	2	113	4
Oligoneuriella rhenana	0	0	0	0	0	0	0	0	0	0	0	0	0	0	0	0	0	1	0	0	0	0	0	0	0	0	0	0	2	0
Orthocladiinae	994	1944	2333	20756	3098	2700	3355	3488	2460	2130	1321	245	325	765	291	3565	1347	1753	2121	1548	410	833	519	1451	3191	1654	35	249	1397	289
Paraleptophlebia	0	0	0	0	0	0	0	0	0	0	0	0	0	3	0	0	0	0	0	0	0	0	0	0	0	0	0	0	0	0
Perla	33	6	0	2	4	1	12	10	2	6	5	28	10	41	8	1	1	0	0	0	0	1	0	1	0	37	43	42	18	15
Perlodes	27	1	0	0	0	1	1	0	0	2	1	0	1	0	0	11	3	1	0	0	1	1	3	0	1	0	3	0	2	0
Philopotamus	0	0	0	0	0	0	0	0	0	0	0	0	0	0	0	0	0	0	0	0	0	0	0	1	0	0	0	4	0	0
Polycelis	0	0	0	0	0	0	0	1	0	0	0	0	0	0	0	0	0	0	0	0	0	0	0	0	0	0	0	0	0	0
Pomatinus	0	0	0	0	0	0	0	0	0	0	0	0	0	0	0	0	0	0	0	0	1	0	0	0	0	0	0	0	0	0
Potamophylax	0	0	0	0	0	0	0	0	0	0	0	0	0	0	0	0	0	0	0	0	0	0	0	0	1	0	0	0	0	0
Protonemura	37	51	30	12	44	8	94	17	201	226	2080	726	162	73	8	20	2	7	4	2	39	6	11	54	97	53	19	52	10	3
Psychodidae	93	23	149	6	4	42	26	8	18	9	9	1	3	3	0	109	17	6	3	3	0	0	0	9	0	9	0	0	2	0
Radix	0	0	0	0	0	0	0	1	0	0	0	0	0	0	0	0	0	0	0	0	0	0	0	0	0	0	0	0	0	0
Rhabdiopteryx	91	37	19	2	3	14	66	213	236	225	1889	6070	755	466	60	1	0	0	0	0	2	0	0	1	1	4	3	2	0	0
Rhithrogena	1404	66	26	243	298	38	113	143	35	32	2434	760	270	691	449	27	43	4	12	17	65	45	28	4	1	247	54	189	406	225
Rhyacophila	52	567	89	307	148	166	57	78	101	79	28	85	43	69	14	13	52	95	53	59	21	39	41	277	165	205	38	44	31	2
Riolus	0	0	0	0	0	0	0	0	0	0	0	0	0	0	3	0	1	0	0	0	0	0	0	0	0	0	0	2	5	0
Sericostoma	0	0	0	0	0	0	0	0	0	0	0	0	0	0	0	0	0	0	0	0	0	0	1	0	0	0	0	0	0	0
Simuliidae	831	9	160	2514	2290	24	328	2862	83	51	86	58	9	269	359	1820	2115	2303	2216	6045	3193	761	1778	10731	2606	1423	2394	2645	4823	821
Stratiomyidae	0	0	0	1	0	0	0	0	0	0	0	0	0	0	0	0	0	3	0	1	1	0	0	0	0	0	0	0	0	0
Syrphidae	0	0	0	0	0	0	0	0	0	1	0	0	0	0	0	0	0	0	0	0	0	0	0	0	0	0	0	0	0	0

	1970s															2010s														
TAXON/SITE	DU1	DU2	DU3	DU4	DU5	DU6	DU7	DU8	GU1	GU2	VE2	VE3	VE4	VE5	VE6	DU1	DU2	DU3	DU4	DU5	DU6	DU7	DU8	GU1	GU2	VE2	VE3	VE4	VE5	VE6
Tabanidae	0	0	2	0	0	0	0	0	0	0	0	0	0	0	0	0	0	0	0	0	0	0	0	0	0	0	0	0	2	0
Taeniopteryx	32	2	0	0	0	0	1	0	0	0	0	8	16	68	6	3	0	1	0	0	0	0	1	0	0	0	0	4	2	0
Tanypodinae	0	2	12	4	3	2	18	6	1	2	0	0	1	1	0	0	0	0	0	1	0	0	0	1	5	0	0	0	0	2
Tanytarsini	53	156	58	293	64	13	189	118	8	12	13	5	1	5	4	108	44	116	89	50	40	175	38	1130	1029	3	2	2	40	22
Thaumaleidae	0	0	0	0	0	0	0	0	0	0	3	0	0	0	0	0	3	0	0	0	0	0	0	2	0	0	0	0	0	0
Tinodes	0	1	0	0	0	0	0	0	0	0	0	0	0	0	0	0	0	0	0	0	0	0	0	0	0	0	0	0	0	0
Tipulidae	6	3	1	8	0	8	1	3	1	5	123	18	13	3	5	0	0	0	0	0	0	0	0	0	1	0	0	0	2	0

Table S1.3. Hydrological parameters calculated in each sampling site.

Code	U	T	Hydrologic index	Definition
Average flow conditions				
MA1	1	D	Mean daily flows	Mean daily flow
MA3	3	D	Variability in daily flows 1	Coefficient of variation in daily flows
MA12-23	1	M	Mean monthly flows	Mean monthly flow for all months (January–December)
MA39	3	M	Variability across monthly flows 2	Coefficient of variation in mean monthly flows
MA41	2	A	Mean annual runoff	Mean annual flow divided by catchment area
MA42	3	A	Variability across annual flows	Variability (calculated as range) in annual flows divided by median annual flows, where variability is calculated as range
Low flow conditions				
ML1-12	1	M	Mean minimum monthly flows	Mean minimum monthly flow for all months (January–December)
ML13	3	M	Variability across minimum monthly flows	Coefficient of variation in minimum monthly flows
ML14	3	A	Mean annual minimum flow	Mean of the lowest annual daily flow divided by median annual daily flow averaged across all years
High flow conditions				
MH1-12	1	M	Mean maximum monthly flows	Mean of maximum monthly flows for all months (January – December)
MH13	3	M	Variability across maximum monthly flows	Coefficient of variation in mean maximum monthly flows
MH14	3	A	Median annual maximum flow	Median of the highest annual daily flow divided by the median annual daily flow averaged across all years

Modified from Olden and Poff (2003). The alphanumeric code refers to the category of the flow regime the hydrologic index was developed to describe, and indices are numbered successively within each category. For example, MA1 is the first index describing the magnitude of average flow conditions. U refers to the units of the index: 1, $m^3\ s^{-1}$; 2, $m^3\ s^{-1}\ km^{-2}$; 3, dimensionless. T refers to the temporal aspect of the hydrograph that the hydrologic index represents: daily (D), monthly (M), or annual (A).

Table S1.4. Absolute values of hydrological parameters for the 1970s (1960–1979) and the 2010s (1996–2015) at each gauging station (FON, BRI, ARG, and EMB in regulated catchment - Upper Durance and STA in free-flowing one - Upper Verdon). Codes: MA1, Mean daily flows; MA3: Variability in daily flows 1; MA12–23: Mean monthly flows (January–December); MA39: Variability across monthly flows; MA41: Mean annual runoff; MA42: Variability across annual flows; ML1–12: Mean minimum monthly flows (January–December); ML13: Variability across minimum monthly flows; ML14: Mean of annual minimum flows; MH1–12: Mean maximum monthly flows (January–December); MH13: Variability across maximum monthly flows; MH14: Median of annual maximum flows.

**Index**	**FON70**	**FON10**	**BRI70**	**BRI10**	**ARG70**	**ARG10**	**EMB70**	**EMB10**	**STA70**	**STA10**
MA1	5.51	4.61	15.31	12.64	28.37	24.85	56.2	49.53	2.71	2.35
MA3	100.74	97.77	86.35	75.28	89.43	74.44	86.42	80.69	103.28	124.19
MA12	1.92	1.76	6.29	6.63	10.38	10.48	21.15	21.7	1.71	2.41
MA13	1.86	1.62	6.07	6.19	9.67	9.31	20.97	19.39	2.45	1.84
MA14	2.23	2.27	6.93	7.89	11.52	13.03	26	28.62	3.96	2.96
MA15	4.72	4.37	13.7	12.19	21.76	22.24	48.45	46.42	5.18	3.44
MA16	13.15	11.79	32.1	28.6	54.8	49.96	116.65	105.68	4.54	3.38
MA17	16.4	12.7	39.05	27.86	74.89	56.79	143.78	117.15	3.01	2.19
MA18	8.56	5.85	23.09	14.97	50.08	37.46	91.2	65.75	1.38	1.29
MA19	4.33	3.09	13.58	10.07	30.43	25.87	52.67	42.43	1.1	0.86
MA20	3.73	3.09	11.24	9.65	23.2	20.79	44.9	37.98	1.3	1.15
MA21	3.63	3.7	10.85	10.77	19.92	20.71	40.77	40.6	2.13	2.2
MA22	3.28	2.93	9.76	9.25	17.11	18.05	37.48	35.34	3.43	3.67
MA23	2.29	2.02	7.43	7.28	11.98	12.68	25.2	25.14	2.18	2.73
MA39	88.73	86.81	78.46	66.84	81.94	67.91	78.89	72.77	76.75	83.97
MA41	0.03	0.02	0.03	0.02	0.03	0.03	0.03	0.02	0.02	0.02
MA42	0.74	0.76	0.83	0.73	0.97	0.78	0.93	0.89	1.54	1.27
ML1	1.71	1.57	5.14	5.05	9.12	8.57	18.55	17.9	0.96	1.43
ML2	1.71	1.5	4.93	4.8	8.72	7.97	18.37	17.33	1.31	1.25
ML3	1.82	1.58	5.39	5.25	9.28	8.55	20.94	19.51	2.03	1.66
ML4	2.63	2.45	7.88	7.53	13.09	13.4	30.17	30.41	3.09	2.08
ML5	6	5.61	16.52	14.68	30.08	28.29	64.77	60.25	3.08	1.98
ML6	9.53	7.66	25.7	18.15	49.17	40.83	98.05	80.93	1.84	1.31
ML7	5.28	3.53	15.81	10.41	35.8	28.27	61.62	47.27	0.92	0.84
ML8	3.26	2.38	10.24	7.83	21.7	19.49	38.44	34.18	0.74	0.57
ML9	2.74	2.09	8.38	7.02	16.67	14.06	31.39	27.09	0.74	0.54
ML10	2.64	2.43	7.76	7.27	13.93	13.29	28.28	27.1	0.89	0.89
ML11	2.34	2.19	6.89	6.61	12.23	12.74	25.54	25.66	1.28	1.27
ML12	1.96	1.71	5.84	5.43	10.44	9.98	21.19	20.61	1.25	1.41

Index	FON70	FON10	BRI70	BRI10	ARG70	ARG10	EMB70	EMB10	STA70	STA10
ML13	71.42	73.29	71.36	58.45	76.1	65.48	72	64.81	77.32	75.1
ML14	0.54	0.51	0.48	0.5	0.44	0.41	0.45	0.42	0.3	0.26
MH1	2.16	2.16	7.06	8.15	11.84	13.69	25.73	30.55	3.53	7.32
MH2	2.06	1.76	7.13	7.05	11.02	10.49	25.94	22.29	6.04	3.61
MH3	3	3.51	8.83	11.14	15.5	19.35	35.84	41.17	9.11	6.19
MH4	9.31	8.66	25.33	21.1	39.92	38	89.69	76.9	9.27	7.26
MH5	27.59	20.59	59.11	48.75	96.67	81.74	196.06	178.42	7.47	6.72
MH6	29.17	21.3	61.35	44.3	118.54	82.66	208.51	181.3	5.16	4.1
MH7	13.97	9.62	34.53	21.73	72.81	53.5	136.72	100.4	2.53	2.74
MH8	6.26	4.66	18.72	12.83	44.21	37.38	81.07	56.59	2.89	1.64
MH9	7.41	7.39	18.07	16.18	46.26	39.16	91.41	75.24	3.93	5.09
MH10	7.09	7.61	17.36	19.09	37.49	41.94	74.11	85.48	6.87	9.47
MH11	6.54	5.08	15.29	13.08	30.87	28.85	81.59	62.57	13.48	13.28
MH12	2.78	2.48	8.83	8.86	14.09	16.34	31.16	33.46	6.19	7.72
MH13	109.15	97.76	91.57	82.14	89.71	73.57	86.77	83.98	118.07	135.56
MH14	9.26	7.81	7.16	5.85	7.79	5.31	6.66	5.8	7.85	13.46

Table S1.5. Variation (%) in hydrological parameters (see Table S1.2 for definition and Table S1.3 for absolute values) between the 1970s (1960–1979) and the 2010s (1996–2015) at each gauging station (FON, BRI, ARG, and EMB in Durance – regulated, and STA in Verdon – free flowing catchment, see Figure 1). Codes: MA1, Mean daily flows; MA3: Variability in daily flows 1; MA12–23: Mean monthly flows (January–December); MA39: Variability across monthly flows; MA41: Mean annual runoff; MA42: Variability across annual flows; ML1–12: Mean minimum monthly flows (January–December); ML13: Variability across minimum monthly flows; ML14: Mean of annual minimum flows; MH1–12: Mean maximum monthly flows (January–December); MH13: Variability across maximum monthly flows; MH14: Median of annual maximum flows;. Increases and decreases greater than 20% are shown in bold letters.

	FON	BRI	ARG	EMB	Average Durance	STA
MA1	-16	-17	-12	-12	-14	-14
MA3	-3	-13	-17	-7	-10	20
MA12	-9	5	1	3	0	**41**
MA13	-13	2	-4	-8	-6	**-25**
MA14	2	14	13	10	10	**-25**
MA15	-7	-11	2	-4	-5	**-34**
MA16	-10	-11	-9	-9	-10	**-26**
MA17	-23	-29	-24	-19	**-24**	**-27**
MA18	-32	-35	-25	-28	**-30**	-6
MA19	-29	-26	-15	-19	**-22**	**-21**
MA20	-17	-14	-10	-15	-14	-12
MA21	2	-1	4	0	1	3
MA22	-11	-5	5	-6	-4	7
MA23	-12	-2	6	0	-2	**25**
MA39	-2	-15	-17	-8	-11	9
MA41	-13	-18	-13	-11	-14	-14
MA42	2	-12	-20	-4	-9	-18

	FON	BRI	ARG	EMB	Average Durance	STA
ML1	-8	-2	-6	-4	-5	**48**
ML2	-12	-3	-9	-6	-8	-5
ML3	-13	-3	-8	-7	-8	-18
ML4	-7	-4	2	1	-2	**-33**
ML5	-6	-11	-6	-7	-8	**-36**
ML6	-20	-29	-17	-17	**-21**	**-29**
ML7	-33	-34	-21	-23	**-28**	-9
ML8	-27	-24	-10	-11	-18	**-23**
ML9	-24	-16	-16	-14	-18	**-27**
ML10	-8	-6	-5	-4	-6	0
ML11	-6	-4	4	0	-2	-1
ML12	-13	-7	-4	-3	-7	13
ML13	3	-18	-14	-10	-10	-3
ML14	-6	4	-7	-5	-4	-14

	FON	BRI	ARG	EMB	Average Durance	STA
MH1	0	16	16	19	13	**108**
MH2	-14	-1	-5	-14	-9	**-40**
MH3	17	26	25	15	**21**	**-32**
MH4	-7	-17	-5	-14	-11	**-22**
MH5	-25	-18	-15	-9	-17	-10
MH6	-27	-28	-30	-13	**-25**	**-21**
MH7	-31	-37	-27	-27	**-31**	9
MH8	-26	-31	-15	-30	**-26**	**-43**
MH9	0	-10	-15	-18	-11	**30**
MH10	7	10	12	15	11	**38**
MH11	-22	-14	-7	-23	-17	-1
MH12	-11	0	16	7	3	**25**
MH13	-10	-10	-18	-3	-10	15
MH14	-16	-18	-32	-13	-20	**71**

Table S1.6. Spearman correlation coefficients between functional traits and the two first axes of the fuzzy correspondence analysis. Significant coefficients ($R > 0.4$; $p < 0.05$) are shown in bold letter.

Functional trait	Categories	Code	Axis 1	Axis 2
Maximal potential size	> 0.25 -0.5 cm	T1_2	**0.98**	-0.13
	> 0.5-1 cm	T1_3	0.27	**0.42**
	> 1-2 cm	T1_4	**-0.96**	0.2
	> 2-4 cm	T1_5	0.06	**-0.64**
	> 4-8 cm	T1_6	-0.03	0.36
Life cycle duration	≤ 1 year	T2_1	-0.02	**-0.69**
	> 1 year	T2_2	0.02	**0.69**
Potential number of cycles per year	< 1	T3_1	**-0.72**	**0.49**
	1	T3_2	**-0.75**	0.14
	> 1	T3_3	**0.79**	-0.25
Aquatic stages	Egg	T4_1	**-0.78**	**0.53**
	Larva	T4_2	**-0.56**	**0.73**
	Nymph	T4_3	**0.72**	**-0.59**
	Adult	T4_4	0.18	-0.13
Reproduction	Ovoviviparity	T5_1	0.18	**-0.64**
	Isolated eggs, free	T5_2	**0.71**	0.34
	Isolated eggs, cemented	T5_3	**-0.9**	**0.44**
	Clutches, cemented or fixed	T5_4	**0.62**	**-0.68**
	Clutches, in vegetation	T5_6	**0.5**	0.32
	Clutches, terrestrial	T5_7	**0.63**	**-0.39**
	Asexual reproduction	T5_8	0.03	-0.13
Dispersal	Aquatic passive	T6_1	**-0.49**	**0.58**
	Aquatic active	T6_2	**-0.9**	0.05
	Aerial passive	T6_3	**0.99**	-0.09
	Aerial active	T6_4	**-0.42**	**-0.67**
Locomotion and substrate relationship	Flier	T21_1	0.28	**0.38**
	Surface swimmer	T21_2	-0.28	**-0.45**
	Full water swimmer	T21_3	-0.02	-0.23
	Crawler	T21_4	**-0.9**	0
	Burrower	T21_5	**-0.57**	0.04
	Interstitial	T21_6	**0.45**	-0.19
	Temporarily attached	T21_7	**0.97**	-0.06
	Permanently attached	T21_8	-0.08	**-0.41**
Resistance forms	Eggs, statoblasts	T7_1	**0.59**	**0.4**
	Cocoons	T7_2	-0.05	-0.16
	Diapause or dormancy	T7_4	**0.86**	0.12
	No resistance forms	T7_5	**-0.77**	-0.28
Food preference	Microorganisms	T8_1	**0.46**	**-0.41**
	Detritus < 1mm	T8_2	**0.84**	0.25
	Dead plant ≥ 1mm	T8_3	**-0.59**	**-0.38**
	Living microphytes	T8_4	0	-0.34
	Living macrophytes	T8_5	**-0.95**	0.26

Functional trait	Categories	Code	Axis 1	Axis 2
Food preference	Dead animal ≥ 1mm	T8_6	**-0.53**	0.08
	Living microinvertebrates	T8_7	**0.92**	-0.16
	Living macroinvertebrates	T8_8	-0.08	**-0.46**
Feeding habits	Deposit feeder	T9_2	-0.4	0
	Shredder	T9_3	**-0.91**	0.09
	Scraper	T9_4	0.23	-0.26
	Filter-feeder	T9_5	**0.97**	-0.06
	Piercer	T9_6	-0.12	**0.64**
	Predator	T9_7	-0.05	**-0.68**
	Parasite	T9_8	0.06	**-0.53**
Respiration	Tegument	T10_1	**-0.76**	0.07
	Gill	T10_2	0.09	-0.31
	Plastron	T10_3	0.3	0.4
	Spiracle	T10_4	**0.85**	0.12

Table S1.7. Results of linear mixed-effect models (LMEs) on biological traits of aquatic invertebrate community. Marginal R^2 (R^2m) and p-values for the whole model and the different terms (date, catchment and the interaction between them) are shown. The sign or trend of the relationship for each term is also displayed, i.e. temporal (Date), spatial (Catchment) and spatiotemporal trends (Date:Catchment). Significant results after applying Bonferroni correction have been highlighted in bold letter (i.e. p-value < 0.001). FC= Free-flowing Catchment (Upper Verdon); RC= Regulated Catchment (Upper Durance). **Results that despite being statistically significant did not meet model assumptions after transformation.

			**Model**		**Date**		**Catchment**		**Date : Catchment**	
**Biological trait**	**Categories**	Code	P – value	R^2m	P – value	Temporal trend (1970s-2010s)	P – value	Spatial trend (greater value)	P – value	Spatio-temporal trend (greater value)
Body size	> 0.25 -0.5 cm	T1_2	**$4.89*10^{-7}$**	**0.63**	**$2.08*10^{-7}$**	+	0.82	=	0.002	=
	> 0.5-1 cm	T1_3	0.58	0.06	0.25	=	0.56	=	0.59	=
	> 1-2 cm	T1_4	**$3.99*10^{-10}$**	**0.77**	**$1.95*10^{-9}$**	-	0.001	=	**$5.85*10^{-5}$**	FC (1970s)
	> 2-4 cm	T1_5	0.165	0.14	0.9	=	0.037	=	0.83	=
	> 4-8 cm	T1_6	0.267	0.24	0.53	=	0.13	=	0.6	=
Life cycle duration	≤ 1 year	T2_1	0.033	0.25	0.59	=	0.005	=	0.97	=
	> 1 year	T2_2	0.038	0.23	0.6	=	0.007	=	0.98	=
Potential number of cycles per year	< 1	T3_1	**$3.5*10^{-4}$**	**0.43**	**0.001**	-	0.032	**=**	0.28	=
	1	T3_2	**$4.38*10^{-4}$**	**0.42**	**$5.45*10^{-4}$**	-	0.068	**=**	0.011	=
	> 1	T3_3	**$1.13*10^{-5}$**	**0.55**	**$2.6*10^{-5}$**	+	0.012	**=**	0.006	=
Aquatic stages	Egg	T4_1	**$1.82*10^{-5}$**	**0.53**	**$1.6*10^{-4}$**	-	0.005	=	0.002	=
	Larva	T4_2	**$1.83*10^{-5}$**	**0.53**	**$8.6*10^{-4}$**	-	**$8*10^{-4}$**	FC	0.003	=
	Nymph	T4_3	**$1.21*10^{-5}$**	**0.55**	**$2.35*10^{-4}$**	+	0.002	=	0.002	=
	Adult	T4_4	0.53	0.06	0.86	=	0.4	=	0.28	=

			Model		Date		Catchment		Date : Catchment	
**Biological trait**	**Categories**	Code	*P* – value	R^2m	*P* – value	Temporal trend (1970s-2010s)	*P* – value	Spatial trend (greater value)	*P* – value	Spatio-temporal trend (greater value)
Reproduction	Ovoviviparity	T5_1	0.098	0.17	0.77	=	0.026	=	0.49	=
	Isolated eggs, free	T5_2	**1.28*10^{-7}**	**0.66**	**5.31*10^{-7}**	+	0.001	=	0.069	=
	Isolated eggs, cemented	T5_3	**1.47*10^{-8}**	**0.71**	**1.09*10^{-7}**	-	**7.05*10^{-4}**	FC	**3.34*10^{-4}**	FC (1970s)
	Clutches, cemented or fixed	T5_4	**2.09*10^{-7}**	**0.65**	**2.31*10^{-6}**	+	**2.6*10^{-4}**	RC	0.017	=
	Clutches, in vegetation	T5_6	**4.87*10^{-6}**	**0.57**	0.008	=	0.013	=	0.008	=
	Clutches, terrestrial	T5_7	0.005	0.32	0.072	=	0.05	=	0.007	=
	Asexual reproduction	T5_8	0.48	0.07	0.75	=	0.23	=	0.53	=
Dispersal	Aquatic passive	T6_1	0.018	0.26	0.016	=	0.068	=	0.56	=
	Aquatic active	T6_2	**7.28*10^{-6}**	**0.56**	**2.86*10^{-6}**	-	0.42	=	0.007	**=**
	Aerial passive	T6_3·	**3.72*10^{-8}**	**0.69**	**1.72*10^{-8}**	+	0.99	=	0.003	**=**
	Aerial active	T6_4	0.04	0.22	0.17	=	0.018	=	0.51	**=**
Locomotion and substrate relationship	Flier	T21_1	**2.54*10^{-10}**	**0.78**	**8.8*10^{-6}**	+	**7.15*10^{-6}**	FC	0.017	**=**
	Surface swimmer	T21_2	0.014	0.26	0.094	=	0.06	=	0.27	**=**
	Full water swimmer	T21_3	0.18	0.16	0.68	=	0.05	=	0.97	**=**
	Crawler	T21_4	**1.87*10^{-5}**	**0.53**	**1.03*10^{-5}**	-	0.24	=	0.004	**=**
	Burrower	T21_5	**6.27*10^{-4}**	**0.41**	**7.66*10^{-4}**	-	0.15	=	0.63	=
	Interstitial	T21_6	0.016	0.27	0.09	=	0.06	=	0.024	=
	Temporarily attached	T21_7	**6.26*10^{-9}**	**0.72**	**3.32*10^{-9}**	+	0.95	=	0.004	=
	Permanently attached	T21_8	0.34	0.12	0.91	=	0.1	=	0.79	**=**
Resistance forms	Eggs, statoblasts	T7_1	**9.71*10^{-5}**	**0.48**	**9.71*10^{-4}**	+	0.08	=	0.98	**=**
	Cocoons	T7_2	0.14	0.13	0.63	=	0.043	=	0.52	**=**
	Diapause or dormancy	T7_4	**6.73*10^{-8}**	**0.68**	**4.52*10^{-8}**	+	0.11	=	0.07	**=**
	No resistance forms	T7_5	**6.05*10^{-7}**	**0.61**	**1.11*10^{-6}**	-	0.024	=	0.1	**=**

			**Model**		**Date**		**Catchment**		**Date : Catchment**	
**Biological trait**	**Categories**	Code	*P* – value	R^2m	*P* – value	Temporal trend (1970s-2010s)	*P* – value	Spatial trend (greater value)	*P* – value	Spatio-temporal trend (greater value)
Food preference	Microorganisms	T8_1	0.09	0.18	0.03	=	0.34	=	0.52	=
	Detritus < 1mm	T8_2	**$3.21*10^{-6}$**	**0.58**	**$4.98*10^{-5}$**	+	0.028	=	0.3	=
	Dead plant ≥ 1mm	T8_3	0.019	0.26	0.038	=	0.043	=	0.11	=
	Living microphytes	T8_4	0.017	0.27	0.5	=	0.038	=	0.04	=
	Living macrophytes	T8_5	**$1.37*10^{-8}$**	**0.72**	**$1.09*10^{-8}$**	-	0.29	=	**$6.5*10^{-4}$**	FC (1970s)
	Dead animal ≥ 1mm	T8_6	0.097	0.17	0.044	=	0.22	=	0.4	=
	Living microinvertebrates	T8_7	**$3.72*10^{-6}$**	**0.58**	**$4.3*10^{-6}$**	+	0.19	=	**$4.8*10^{-4}$**	FC (2010s)
	Living macroinvertebrates	T8_8	0.67	0.05	0.87	=	0.32	=	0.66	=
Feeding habits	Deposit feeder	T9_2	0.085	0.17	0.05	=	0.24	=	0.85	=
	Shredder	T9_3	**$5.12*10^{-7}$**	**0.63**	**$3.82*10^{-7}$**	-	0.16	=	0.001	=
	Scraper	T9_4	0.041	0.22	0.11	=	0.05	=	0.18	=
	Filter-feeder	T9_5	**$1.09*10^{-8}$**	**0.71**	**$5.45*10^{-9}$**	+	0.99	=	0.004	=
	Piercer**	T9_6	0.002	0.45	0.96	=	**$5.3*10^{-4}$**	FC	0.49	=
	Predator	T9_7	0.19	0.14	0.97	=	0.06	=	0.5	=
	Parasite	T9_8	0.005	0.32	0.33	=	0.007	=	0.12	=
Respiration	Tegument	T10_1	**$1*10^{-6}$**	**0.61**	**$3.81*10^{-7}$**	-	0.45	=	0.1	=
	Gill	T10_2	0.22	0.12	0.07	=	0.54	=	0.69	=
	Plastron**	T10_3	**$1.27*10^{-11}$**	**0.82**	**$6.21*10^{-6}$**	+	**$7.1*10^{-7}$**	FC	0.01	=
	Spiracle	T10_4	**$5.28*10^{-9}$**	**0.73**	**$4.22*10^{-9}$**	+	0.15	=	0.06	=

Figure S1.1. Boxplots showing the evolution of climatic variables from the 1970s (1960–1979) to the 2010s (1996–2015). Horizontal lines represent the median, 25th and 75th quartile values. Significance (*p*-values, *F*-value and goodness of fit-R^2) of the variation of each climatic variable between both periods according to linear-mixed effect models (LMEs) is shown.

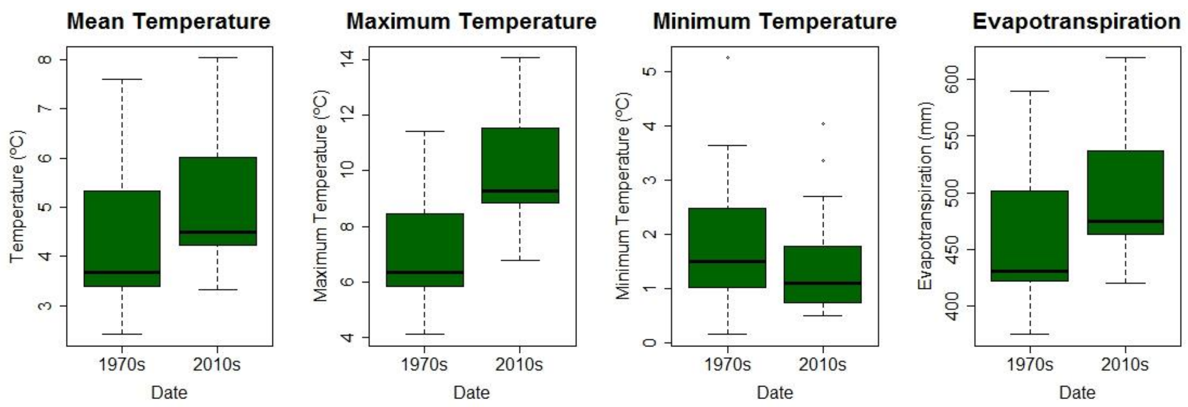

$p < 0.001$; F=146.17; R^2=0.99 $p < 0.001$; F=909.19; R^2=0.99 $p < 0.01$; F=16.82; R^2=0.76 $p < 0.001$; F=340.5; R^2=0.99

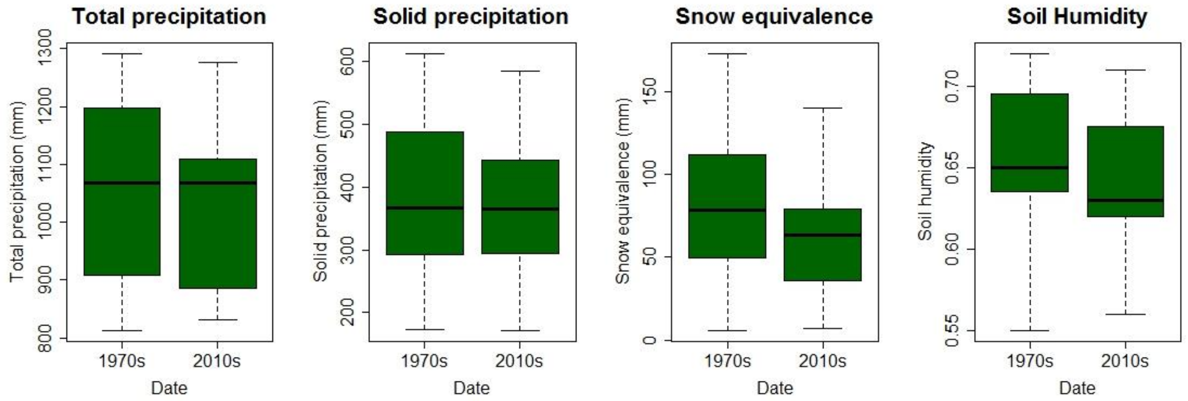

$p < 0.05$; F=8.54; R^2=0.41 Non-significant $p < 0.01$; F=16.65; R^2=0.94 $p < 0.001$; F=50.55; R^2=0.94

Figure S1.2. Mann-Whitney U results to check the climatic representativeness of the years with biological samples with respect to the whole 20 year period in the 1970s (1960-1979) and the 2010s (1996-2015). On the X-axis, 1 and 0 refer to years with and without biological samples, respectively.

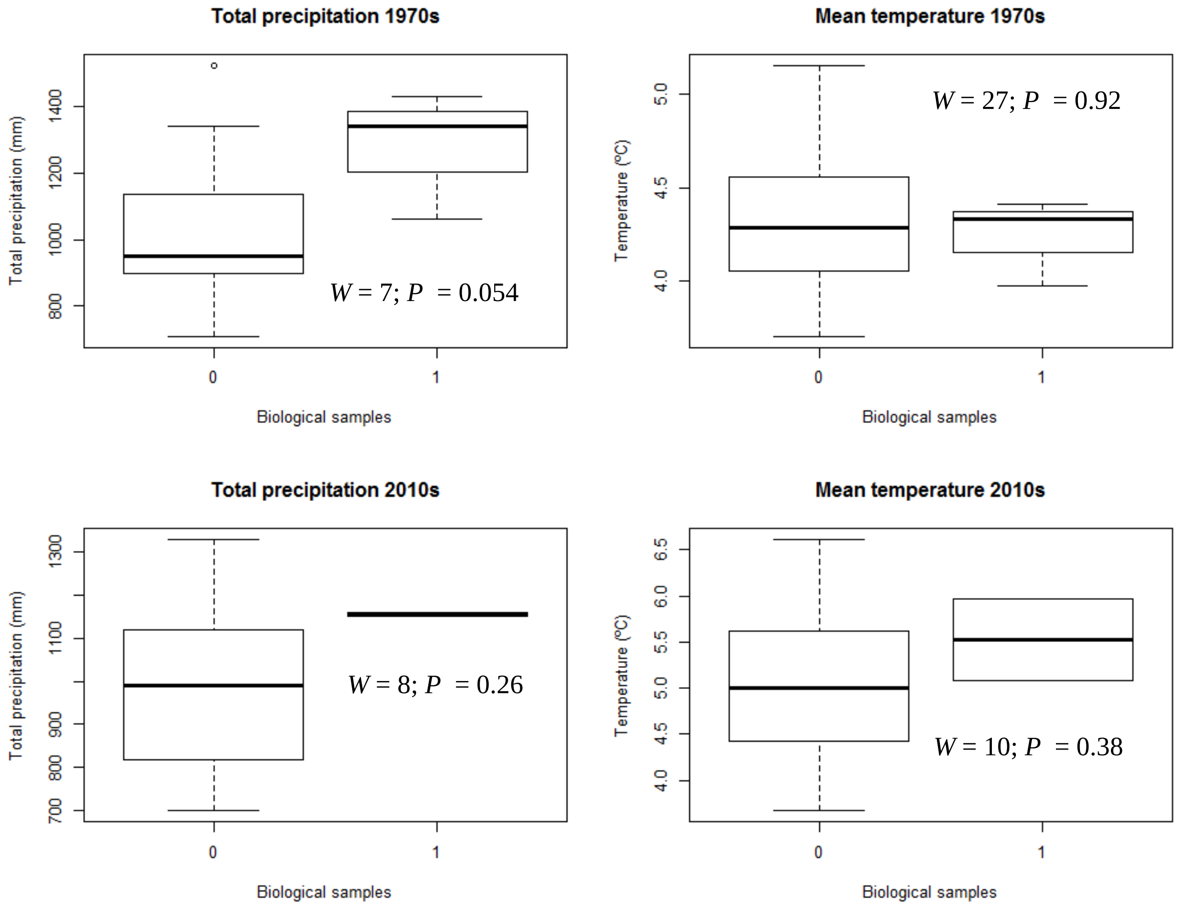

Figure S1.3. Results of null models comparing γ-diversity estimated for free-flowing catchment (dashed lines that represent empirical values) and for an equivalent number of randomly selected sites from regulated catchment (histogram values that represent the simulated distributions) in the 1970s and the 2010s. *Z*-scores and *p*-values are shown.

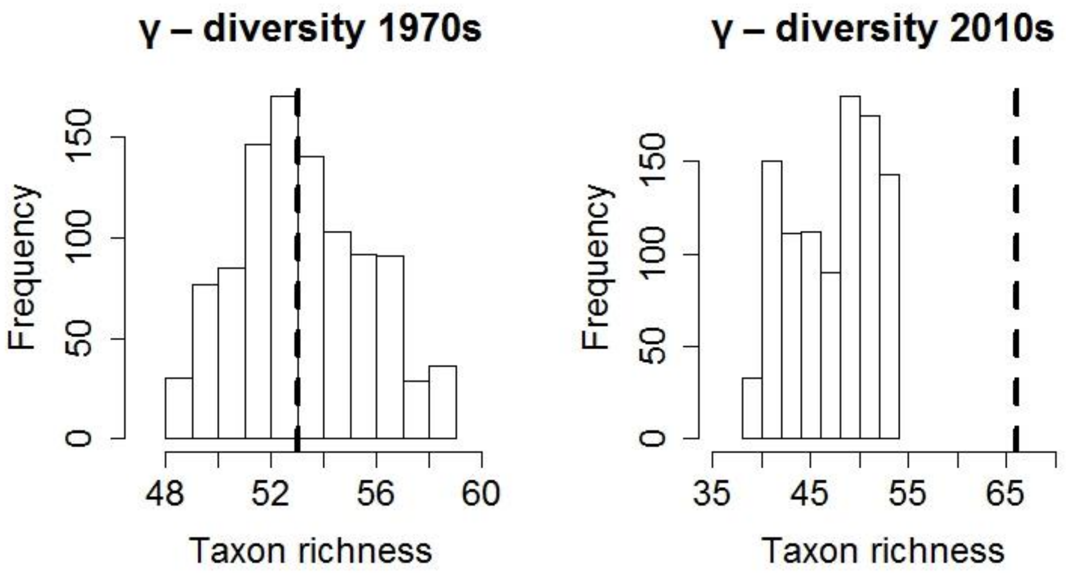

Z – score = -0.22
P – value = 1

Z – score = 4.35
P – value = 0.001

Figure S1.4. Boxplots showing the observed (mean) differences in psychrophilic and thermophilic taxa between the 1970s and the 2010s in regulated (Durance catchment, red colour) and free-flowing (Verdon catchment, blue colour) catchments. Significances (*p* – values) of the different terms included in linear mixed-effect models (LMEs) are also shown. ns. = non-significant

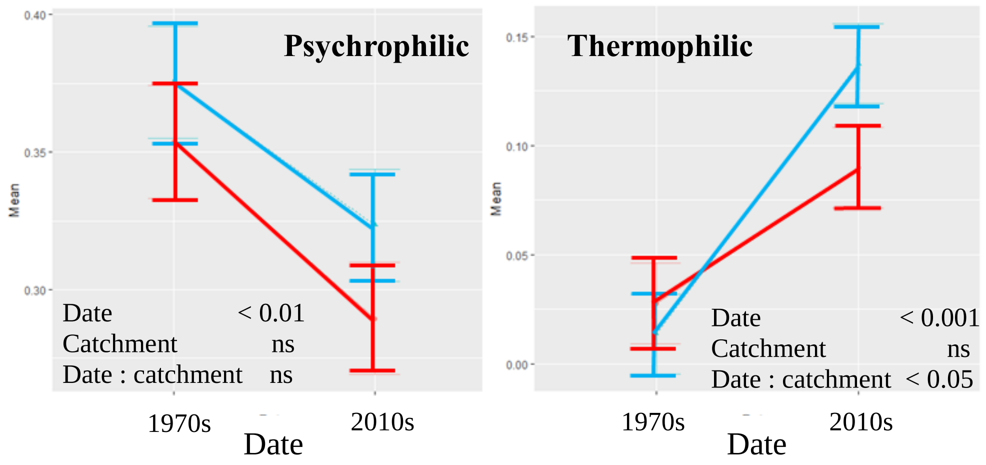

Figure S1.5. Comparison of compositional patterns for regulated (i.e. DUR, red color) and free-flowing (i.e. VER, blue color) catchments between the 1970s and the 2010s. Non-metric multidimensional scaling (NMDS) for the a) 1970s and b) 2010s, c) Procrustes analyses comparing the position of each site in the multivariate space through time (note that the label of the sampling site represents 2010s and the points represent the 1970s) and d) Multivariate dispersion.

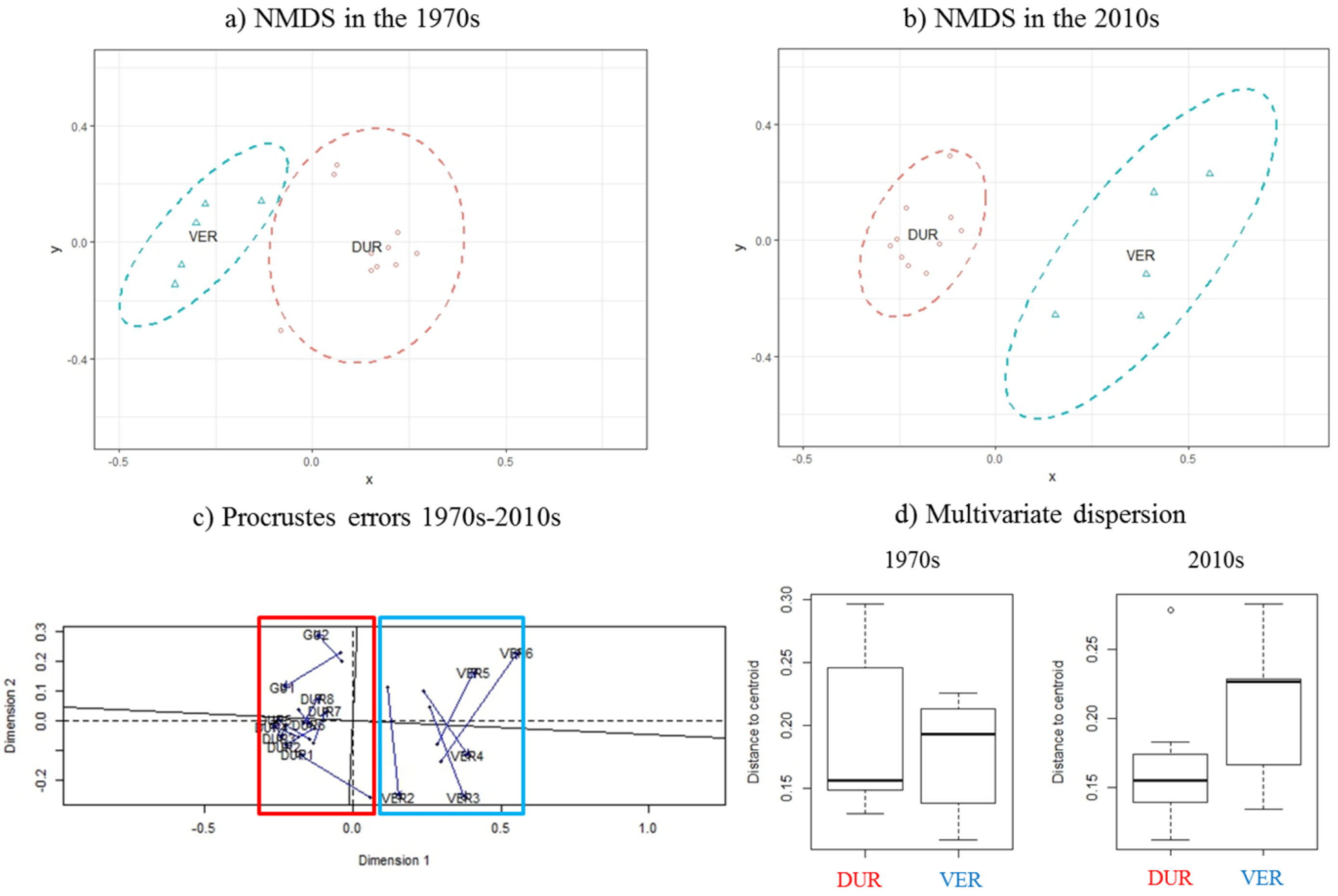

Figure S1.6. Results of non-metric multidimensional scaling (NMDS) for aquatic communities in regulated (DUR - red colour) and free-flowing catchments (VER - blue colour) accounting for the different seasons (summer and winter) in the 1970s and the 2010s.

1970s **2010s**

Summer

Winter

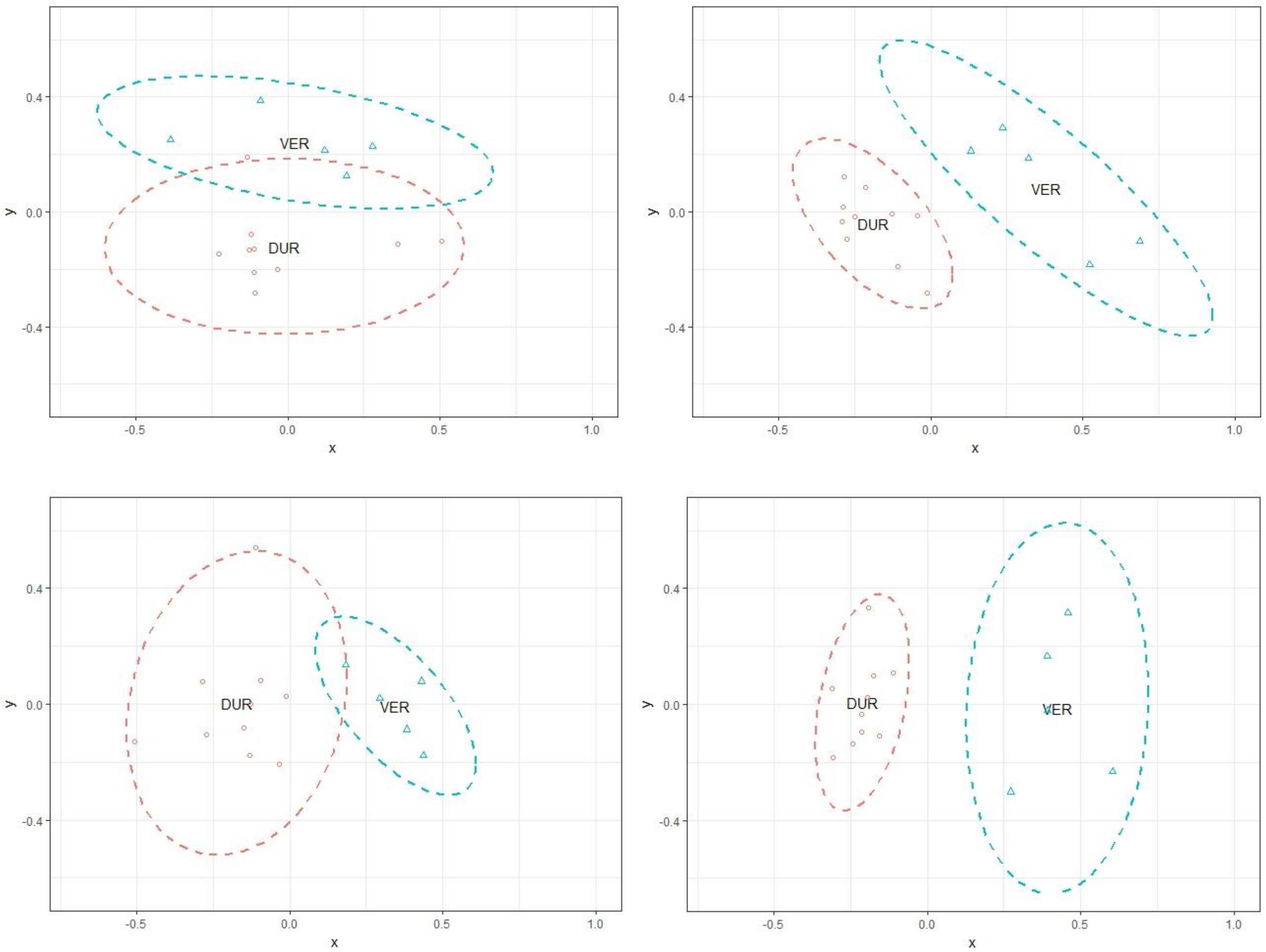

Supplement: Supplementary file 1 [file GCB-25-1612-s001.pdf]
